# Predicting folding pathways between RNA conformational structures guided by RNA stacks

**DOI:** 10.1186/1471-2105-13-S3-S5

**Published:** 2012-03-21

**Authors:** Yuan Li, Shaojie Zhang

**Affiliations:** 1 Department of Electrical Engineering and Computer Science, University of Central Florida, Orlando, FL, 32816-2362, USA

## Abstract

**Background:**

Accurately predicting low energy barrier folding pathways between conformational secondary structures of an RNA molecule can provide valuable information for understanding its catalytic and regulatory functions. Most existing heuristic algorithms guide the construction of folding pathways by free energies of intermediate structures in the next move during the folding. However due to the size and ruggedness of RNA energy landscape, energy-guided search can become trapped in local optima.

**Results:**

In this paper, we propose an algorithm that guides the construction of folding pathways through the formation and destruction of RNA stacks. Guiding the construction of folding pathways by coarse grained movements of RNA stacks can help reduce the search space and make it easier to jump out of local optima. RNAEAPath is able to find lower energy barrier folding pathways between secondary structures of conformational switches and outperforms the existing heuristic algorithms in most test cases.

**Conclusions:**

RNAEAPath provides an alternate approach for predicting low-barrier folding pathways between RNA conformational secondary structures. The source code of RNAEAPath and the test data sets are available at http://genome.ucf.edu/RNAEAPath.

## Introduction

RNA molecules play critical roles in the cell. The secondary structures of RNA molecules have been extensively studied because they provide insights into the functionality of RNAs. Native (functional) RNA secondary structures are usually thermodynamically stable and many of them are also the minimum free energy (MFE) structures. Nevertheless, at times, RNA molecules may fold into alternative secondary structures in order to participate in certain biological processes. For example, the SV-11 RNA folds into a metastable conformational structure and acts as a template for its own replication using Q*β *replicase [1,2]. Further, RNA conformational switches can transform between alternative secondary structures dynamically in response to various environmental stimuli (such as heat shock and cold shock) [3-6], and carry out RNA-mediated biological activities, such as switching on or off downstream gene translation activities [7-9], regulating RNA splicing via multiple-state splicesomal conformations [10], and regulating the life cycles of virus [11].

The conformational transformations between alternative structures involve the folding of an RNA molecule into a series of sequential adjacent intermediate structures [12]. RNA folding pathways provide valuable information for understanding the catalytic and regulatory functions of RNAs (such as hok/sok of plasmid R1 [13]). RNA folding pathways may also impact subsequence biological events (such as formation of tertiary structures). Furthermore, prediction algorithms can help the design of RNA switches by providing prescribed structural alternatives.

In this paper, we present a new approach, RNAEAPath, for computing near optimal direct or indirect folding pathways between two secondary structures of an RNA molecule. We guide the search for low energy barrier folding pathways by integrating a variety of strategies for simulating the formation and destruction of RNA stacks in a flexible framework. Benchmark tests on conformational switches show that RNAEAPath produces lower energy barrier folding pathways and outperforms the existing heuristic approaches in most test cases.

### Preliminary

Consider an RNA sequence as a string *x *= *x*_1 _... *x_n _*of *n *letters over alphabet ∑ = {A, U, G, C}. A pair of complementary nucleotides *x_i _*and *x_j_*, can form hydrogen bonds and interact with each other, denoted by *x_i _*· *x_j_*. In this paper, we only consider the canonical base pairings (*A *· *U *and G · C) and the wobble base pairing (G · U). A *secondary structure S *of the RNA sequence *x *is a set of disjoint paired bases (*i*, *j*), where 1 ≤ *i *<*j *≤ *n*. *S *may be represented by a length *n *string of dots and brackets, where dots represent unpaired bases and brackets represent paired bases. An RNA structure can comprise of *stacks *which are lists of consecutive base pairs ({(*i*, *j*), (*i *+ 1, *j *- 1), . . . , (*i *+ *w*, *j *- *w*)} such that *x_i _*· *x_j_*, . . . , *x_i+w _*· *x_j-w_*), and unstacking base pairs. A secondary structure is *pseudoknotted *if it contains two base pairs (*i*, *j*) and (*i*', *j*') with *i *<*i*' <*j *<*j*'. In this paper, we only consider pseudoknot-free structures. A base pair is compatible with a secondary structure if the base pair can be added to the structure without leading to a pseudoknotted structure or pairing a base with more than one partner. A stack is compatible with *S *if each base pair in the stack is either in *S *or is compatible with *S*.

The free energy of a secondary structure *S *is denoted by *E*(*S*). The set of *neighboring structures *of *S *consists of all structures that differ from *S *by an addition or deletion of exactly one base pair. For two secondary structures *A *and *B*, the *distance *between *A *and *B *is the number of base pairs in *A *not in *B *plus the number of base pairs in *B *not in *A *(i.e. |(*A *- *B*) ∪ (*B *- *A*)|). A *folding pathway *from *A *to *B *is a sequence of intermediate structures *A *= *S*_0_, . . . , *S_m _*= *B *such that for all 0 ≤ *i *<*m*, intermediate structure *S_i_*_+1 _is a neighboring structure of *S_i_*. A folding pathway is *direct *if the intermediate structures contain only base pairs in *A *and *B *(i.e. *S_i _*⊆ *A *∪ *B *for 1 ≤ *i *<*m*) and otherwise is *indirect*. The *saddle point *of a pathway is an intermediate structure with the highest energy, and the *energy barrier *of a pathway is the energy difference between its saddle point and the initial structure. Since the folding of RNA structures is thermodynamically-driven and tends to avoid high-energy intermediate structures, current computational methods aim to find RNA folding pathways with the lowest energy barriers.

### Previous studies

A lot of research has been done on predicting low energy barrier folding pathways. Morgan and Higgs proposed a greedy algorithm that employs the Nussinov model [14,15] for computing direct folding pathways with minimum energy barrier. They also described a heuristic that samples low energy structures from the partition function and glues them together by direct pathways [16]. The Nussinov model is simple and easy to implement, in which base stacking and loop entropies have no energetic contributions. Based on this model, Thachuk *et al*. [17] developed an exact algorithm, PathwayHunter, which exploits elegant properties of bipartite graphs for finding the globally optimal direct pathways. However, the Nussinov model is not as accurate as the Turner energy model [18,19] for approximating RNA thermodynamics. An exact solution based on the Turner energy model is also available. BARRIERS [20,21], exactly computes the globally optimal folding pathways between any two locally optimal secondary structures. BARRIERS reads an energy sorted list of RNA secondary structural conformations produced by RNAsubopt [22] and is able to compute both direct and indirect low energy barrier pathways.

Nevertheless, the above exact solutions are all exponential in time, because the problem itself is NP-hard [23]. Many heuristic algorithms have also been proposed following the seminal work of Morgan and Higgs. Flamm *et al*. [24] used breadth-first search in their heuristics (in Vienna RNA Package [25]) and kept the best *k *candidates at each step to bound the search. Voss *et al*. [26] devised a straightforward strategy for greedily searching direct pathways. Geis *et al*. [27] described a greedy heuristic to explore the search space of direct pathways and they also integrated look ahead techniques to diminish the search space. Recently, Dotu *et al*. [28] developed RNATabuPath, a fast heuristic that employs a TABU semi-greedy search to construct near optimal (both direct and indirect) folding trajectories. In addition, other heuristic approaches, by splitting the pathways into shorter pathways and solving each individually, have also been proposed [29,30]. There are also other formula presented for the prediction of RNA folding kinetics (see Flamm and Hofacker's review [31] for a systematic discussion).

Many of the existing heuristic algorithms start from an initial structure *A*, and, at each single step *i*, walk from the intermediate structure *S_i _*to one of its neighbors *S_i_*_+1 _until finally the end structure *B *is reached. The definition of neighborhood relationships as well as the fitness functions can be different. The *fitness function *of *S_i _*is usually defined on the free energy of *S_i_*, or the distance from *S_i _*to *B*, or a function of both. In general, greedy algorithms select the 'best' neighbor structure that has the best fitness. In contrast, semi-greedy algorithms may select any one from the top *k *structures for randomization. RNATabuPath, which is more sophisticated and outperforms other methods [28], keeps a tabu list for saving recently taken moves such that they can not be applied in certain steps until being removed from the tabu list. In general, during the construction of a folding pathway, these heuristic algorithms select the next intermediate structures from a set of neighboring structures that have the top lowest free energy or have the top shortest distance to *B *(or the combination of both).

### Motivations

However, using energy to guide the construction of folding pathways in the above-mentioned heuristic algorithms has its downsides. The RNA energy landscapes can be extremely large and rugged [11,32] and the ruggedness of RNA energy landscape may cause the energy-guided search to become trapped in a local optimum. Similar to using structural rearrangements for modeling RNA folding kinetics [33], we want to construct candidate folding pathways in a manner that make it easier to jump out of local optima. It has been revealed that stacking base pairs contribute significantly to the stabilization of RNA secondary structures [34,35]. The dominant RNA folding pathways involve the formation and destruction of the stacks, and the cooperative formation of a stack along with the partial melting of an incompatible stack [36]. In this paper, we propose to guide the construction of pathways by the formation and destruction of stacks (not by free energy or by distance to the end structure). We still select the constructed folding pathways according to their energy barriers. Although the construction of folding pathways is not driven by thermodynamics, the selection of folding pathways is based on energy barriers. Guiding the construction of folding pathways by coarse grained movements of RNA stacks may help reduce the search space and makes it easier to jump out of local optima. In the rest of this paper, the Methods section describes the representation of folding pathways and the detailed strategies employed by RNAEAPath. The Results and Discussion section presents benchmarking results of RNAEAPath against existing methods followed by concluding remarks in the Conclusions section.

## Methods

### Representation of RNA folding pathways

Given an initial structure *A *and an end structure *B*, we use a sequence of *actions *successively applied to *A*, rather than a sequence of intermediate structures, to represent a folding pathway from *A *to *B*. Representing a pathway by an action chain can avoid cyclic additions and deletions of base pairs and make it easy to simulate the formation and deletion of RNA stacks. A similar representation has also been employed in the previous work of Thachuk *et al*. [17].

We use two types of actions, add*_i,j _*and del*_i,j _*in the representation of RNA folding pathways. For an intermediate secondary structure *S *of an RNA sequence *x*, the action add*_i,j _*denotes the 'add'ition of base pair (*i*, *j*) to *S *(i.e. add*_i,j_*(*S*) = *S *∪ {(*i*, *j*)}) and del*_i,j _*denotes the 'del'etion of base pair (*i*, *j*) from *S *(i.e. del*_i,j_*(*S*) = *S *- {(*i*, *j*)}). An action is *direct *if it concerns a base pair in *A *∪ *B *and *indirect *otherwise. The simplest direct pathways from *A *to *B *concern sequential deletions of all base pairs in *A *- *B *followed by additions of all base pairs in *B *- *A*.

**Figure 1:**
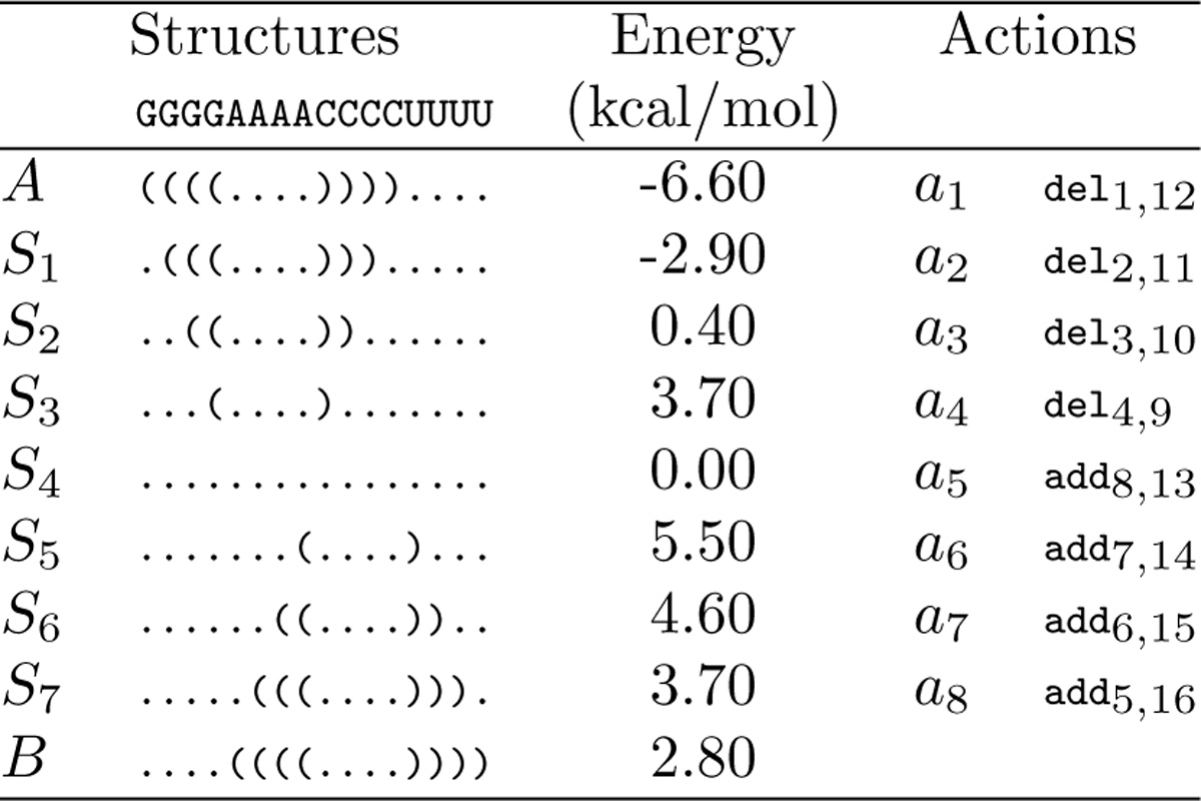
**A simple folding pathway that converts an RNA sequence from structure ***A ***to *B.*** A simple folding pathway that converts an RNA sequence from structure *A *to *B*. The leftmost column shows a simple direct pathway from *A *to *B*, the center column shows the free energies (in kcal/mol) of the intermediate structures, and the rightmost column presents the action chain *a*_1_, . . . , *a*_8 _for this pathway.

Consider an example sequence *x *= GGGGAAAACCCCUUUU with initial and final structures shown in Figure 1. This simple pathway is obtained by first deleting all GC pairs from *A *until the RNA is single stranded, and then adding all AU pairs until *B *is obtained. Note that each intermediate structure *S_i _*differs from both its successor and predecessor by exactly one base pair. The actions in the example are all direct actions and the energy barrier is 5.50 - (-6.60) = 12.10 kcal/mol.

An addition action add*_i,j_*(*S*) *conflicts with S *if either *x_i _*or *x_j _*is already paired in *S*, and it *clashes with S *if there exists a base pair $\left\{\left(x_{i}^{\prime },x_{j}^{\prime }\right)\in S|i<i^{\prime }<j<j^{\prime }\text{ or }i^{\prime }<i<j^{\prime }<j\right\}$. A deletion action del*_i,j_*(*S*) *conflicts with S *if (*x_i_*, *x_j_*) ∉ *S*. An addition or deletion action is *valid *and can be applied to *S properly *if it neither conflicts with nor clashes with *S*.

A pathway from *A *to *B *can be represented by an *action chain*, which is a sequence of valid actions *a*_1_, . . . , *a_m _*such that *S*_0 _= *A*, *S_t _*= *a_t_*(*S_t_*_-1_) for 1 ≤ *t *≤ *m *and *S_m _*= *B*. Note that an action chain for *A *to *B *implies a sequence of valid actions that can be successively applied to *A *without introducing conflicts or clashes and produce *B*. We use the term "action chain" when the sequence is certified to be valid, and the term "sequence of actions" if its validity is not guaranteed.

This representation of a pathway *p *from *A *to *B *has the following important properties. First, every folding pathway can be represented by a unique action chain and every action chain represents a unique folding pathway (note that it is not necessarily true for a sequence of actions). Second, rearranging the order of actions in *p *results in a new sequence of actions which represents a new folding pathway from *A *to *B *when it is *valid*. (It is an action chain that can be successively applied to *A *properly and obtain *B*.) Third, introducing a pair of complementary actions (e.g. add*_i,j _*and del*_i,j_*) to *p *results in a new sequence of actions which also represents a new folding pathway from *A *to *B *if it is *valid*.

In RNAEAPath, folding pathways are represented in the form of action chains, instead of a sequence of intermediate structures. This representation makes the life cycle of a folding pathway transparent to the algorithm and also makes it easier for us to simulate the cooperative formation and destruction of RNA stacks by re-arranging the order of actions or introducing multiple pairs of complementary actions.

### Predicting low energy barrier folding pathways

**Figure 2:**
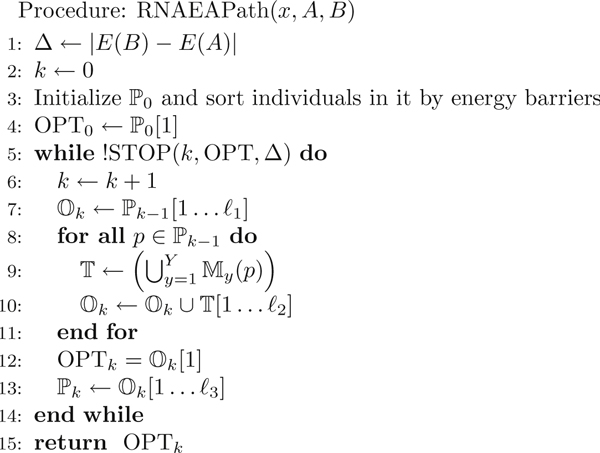
**Overview of RNAEAPath.** Overview of RNAEAPath. In this procedure, the input is an RNA sequence *x *with the start and end structures *A *and *B*, and the output is the best folding pathway in *k *iterations (OPT*_k_*). For notations, *k *is the number of iterations, ℙ_0 _is the initial population, and ℙ _k _is the folding pathway population of the *k^th ^*iteration. T contains all the offspring folding pathways produced by applying mutation strategies $M_{1},\ldots ,M_{Y}$ to each pathway *p *in the (*k *- 1)*^st ^*population. $O_{k}$ is an ordered list of offspring folding pathways of the *k^th ^*generation, from which, the population for the next iteration ( ℙ*_k_*_+1_) is selected. Folding pathways in ℙ*_k _*and $O_{k}$ are sorted based on their fitness and ℙ*_k_*[1 . . . *ℓ*] are the top *ℓ *best folding pathways in ℙ*_k_*.

Given an RNA sequence *x*, an initial structure *A *and a final structure *B*, RNAEAPath computes a near optimal low energy barrier folding pathway from *A *to *B *in an evolutionary algorithm framework [37]. Figure 2 elucidates the overall paradigm for RNAEAPath. In this algorithm, the population of each generation is comprised of folding pathways ordered by their *fitness*. The functions $M_{y}\left(p\right)$ are *mutation strategies*, each of which takes in a pathway *p *and produces a set of offspring pathways. These mutation strategies are central to the effectiveness of RNAEAPath and will be discussed in the *Mutation strategies *subsection. *ℓ*_1_, *ℓ*_2_, *ℓ*_3_, *MAX *and *γ *are positive integer control parameters.

The initial population of RNAEAPath, ℙ_0_, is filled with a set of simple pathways. Then, the algorithm goes through several iterations. ℙ*_k_*_-1_ is the population of the *k *- 1*^st ^*iteration. In the *k^th ^*iteration, the algorithm produces $O_{k}$ (an ordered list of pathways) and ℙ*_k _*(the population of the *k^th ^*iteration) from ℙ*_k_*_-1_. $O_{k}$ stores the best *ℓ*_1 _pathways in ℙ*_k_*_-1 _and the best *ℓ*_2 _pathways produced by each *p *∈ ℙ*_k_*_-1_. More specifically, each pathway *p *∈ ℙ*_k_*_-1 _produces $t_{y}^{k}$ offsprings through every mutation strategy $M_{y}\left(1\leq y\leq Y\right)$. The resulting offsprings produced by *p *are stored in a temporary list T, and the top *ℓ*_2 _pathways are added to $O_{k}$. Finally, the best solution of the *k^th ^*iteration, termed as OPT*_k_*, is the best pathway in $O_{k}$. And, ℙ*_k _*(the population of the *k^th ^*iteration) is composed of the best *ℓ*_3 _pathways of $O_{k}$ and will be used in the next iteration to produce ℙ*_k_*_+1_. This helps keep the diversity of the population large, since ℙ*_k _*contains at most *ℓ*_2 _offsprings produced by each *p *∈ ℙ*_k_*_-1_, no matter how many high-qualified offsprings are produced by each pathway. The algorithm terminates when a stopping condition is met, and it returns the best solution of the last iteration. Since $O_{k}$ retains the best *ℓ*_1 _pathways from ℙ*_k_*_-1 _in each iteration, the best one ever encountered by the algorithm is retained in lists $O_{k}$ and ℙ*_k_*, and stored in OPT*_k_*. So, OPT*_k _*has no worse fitness when compared to OPT*_k_*_-1_, and RNAEAPath always returns the best action chain it ever discovered.

In the remaining of this section, we discuss details regarding fitness evaluation, initialization of the population, stopping conditions and mutation strategies of RNAEAPath.

#### Fitness of action chains

The order of folding pathways (valid action chains) is primarily determined by their energy barriers. In case of a tie, the order is determined by the average of energy differences between the initial structure *A *and intermediate structures. Note that lower energies are preferred in the previous two methods of ordering. If a tie still exists, then shorter action chains are preferred. Action chains are ordered arbitrarily if their relative order can not be determined based on these three criteria.

#### The initial population of folding pathways

**Figure 3:**
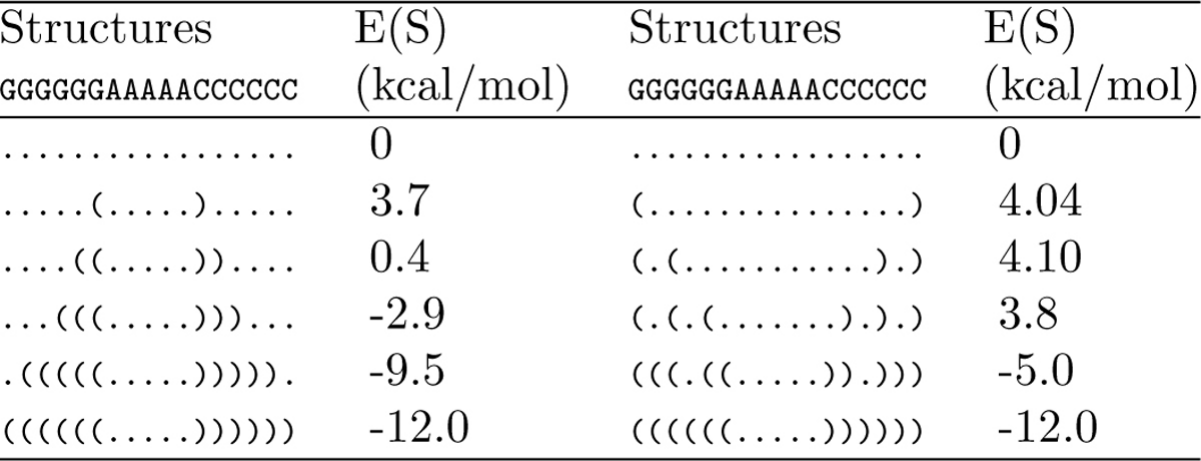
**Two different folding pathways that form an identical stack.** Two different folding pathways that form an identical stack. Left: The stack is formed successively. Right: The stack is constructed by random formation of base pairs. The right pathway yields a higher energy barrier because the randomly introduced base pairs form unpaired loop regions that result in additional entropic penalties.

The initial population, ℙ_0_, contains 4 *simple *pathways from *A *to *B *formed by first deleting all base pairs in *A *- *B *and then adding those in *B *- *A*, similar to the pathway shown in Figure 1. Although we can also arrange base pair deletions and additions in an arbitrary order, we tailor them in a manner that simulates successive degradation and formation of RNA stacks. This is because random deletions and additions of base pairs tend to form additional unpaired loop regions that introduce entropic penalties (see Figure 3 for an illustration). We can degrade or form each stack either from the outmost base pair to the innermost base pair or vice verse. Usually, it yields a lower energy barrier if we degrade a stack from the outmost base pair to the innermost base pair and form a stack from the innermost base pair to the outmost base pair. However, for the sake of simplicity and generosity, we construct 4 simple pathways in ℙ_0_, which degrade all the stacks from the same direction and form all the stacks from the same direction. These simple pathways constitute a diversified and unbiased initial population for the algorithm to start from.

#### The number of offsprings produced by each mutation strategy

In each generation, the expected total number of offsprings produced by each individual is a constant positive integer L. The number of offsprings that each individual produces using mutation strategy $M_{y},\left(1\leq y\leq Y\right)$, in the *k^th ^*generation, is denoted by $\ell_{M_{y}}^{k}$. In the initial generation, $\ell_{M_{y}}^{0}$ is equivalent to L/*Y *for all the mutation strategies. In the *k^th ^*generation, $\ell_{M_{y}}^{k}$ is determined adaptively according to the quality of the offsprings produced using $M_{y}$ in the *k *- 1*^st ^*iteration. Let $b_{M_{y}}^{k-1}$ be number of offsprings that are both produced through $M_{y}$ and selected to construct ℙ_*k*-1_, the population of the *k *- 1*^st ^*generation. Then, $\ell_{M_{y}}^{k}$ in the *k^th ^*generation is computed as follows.

$$\ell_{M_{y}}^{k}=\max\left\{\begin{array}{c} L_{\min}\\ \;\\ \frac{\left(b_{y}^{k-1}/\ell_{M_{y}}^{k-1}\right)}{\overset{Y}{\underset{y^{\prime }=1}{\sum }}\left(b_{y^{\prime }}^{k-1}/\ell_{M_{y^{\prime }}}^{k-1}\right)}L \end{array}\right)$$

Mutation strategies that have produced more high quality offsprings in the (*k *- 1)*^st ^*iteration are allowed to generate more offsprings in the *k^th ^*generation. In contrast, mutation strategies that perform poorly in the *k *- 1*^st ^*generation, are only allowed to generate a small number (L*_min_*, with default value 3) of offsprings. Note that, the sum of $\ell_{M_{y}}^{k}$ for 1 ≤ *y *≤ *Y *may be greater than *ℓ*.

#### Stopping conditions

The algorithm terminates when (1) the current best solution achieves the lowest possible value |*E*(*B*) - *E*(*A*)|, or (2) when no improvement has been found over *γ *consecutive iterations (a plateau), or (3) when *MAX *number of iterations have passed and successive iterations do not discover better results. Note that the algorithm may simulate further than *MAX *iterations if improvements are made in the very last iteration and it stops immediately if no improvement is made between successive iterations. More specifically, the algorithm stops when any of the following conditions is satisfied:

1. the energy barrier of OPT*_k _*is equivalent to |*E*(*B*) - *E*(*A*)|.

2. *k *>*γ *and the fitness of OPT*_k _*is equivalent to that of OPT*_k_*_-_*_γ_*.

3. *k *≥ *MAX *and the fitness of OPT*_k _*is equivalent to that of OPT*_k_*_-1_.

### Mutation strategies

In RNAEAPath, the mutation strategies employed to evolve folding pathways can be categorized into three types: (1) rearranging the order of actions, (2) introducing indirect pathways and (3) formation of a single stack or cooperative conversion of a pair of incompatible stacks. In this section, let $M_{1},\ldots ,M_{Y}$ denote the mutation strategies and let *p *= *a*_1_, . . . , *a_m _*denote the input pathway *A *= *S*_0_, . . . , *S_m _*= *B*. For each mutation strategy $M_{y}\left(p\right)$, we describe the process for generating one new pathway *q *using each mutation strategy when given *p*.

#### Type 1: reordering of actions

As described in the subsection of representation of RNA folding pathways, shuffling the order of actions of the input pathway *p *can result in a new pathway from *A *to *B*. In RNAEAPath, two mutation strategies of this type are employed. $M_{1}$ changes the position of an arbitrary action, and $M_{2}$ swaps the positions of two arbitrary actions.

M_1_: Let $M_{1}^{t_{1},t_{2}}\left(p\right)$ denote the sequence of actions obtained by first removing an action $a_{t_{1}}$ (1 ≤ *t*_1 _≤ *m*) from *p *and then inserting it after $a_{t_{2}}$, for all *t*_2 _∈ {0,..., *t*_1 _- 1, *t*_1 _+ 1, . . . , *m*}. Note that the resulting sequence of actions may not necessarily be a valid action chain. For instance, in Figure 1, $M_{1}^{1,4}\left(p\right)=a_{2},a_{3},a_{4},a_{1},a_{5},\ldots ,a_{8}$ and $M_{1}^{3,2}\left(p\right)=p$ are valid action chains, while $M_{1}^{8,1}\left(p\right)=a_{1},a_{8},a_{2},\ldots ,a_{7}$ is not.

The procedure for computing $M_{1}^{t_{1},t_{2}}\left(p\right)$ is described in the following.

1. Choose *t*_1 _uniformly at random from the interval [1, *m*].

2. Compute the interval [*l*, *u*], (*t*_1 _<*l *<*u *<*m*), where *l *is the minimum and *u *is the maximum such that for all *t*_2 _∈ [*l*, *u*] and *t*_2 _≠ *t*_1_, $M_{1}^{t_{1},t_{2}}\left(p\right)$ is a valid action chain.

3. Choose *t*_2 _from the interval [*l*, *u*].

3.1. If $a_{t_{1}}$ is an addition operation, for all *l *≤ *t *<*t*' ≤ *u *and *t *≠ *t*' ≠ *t*_1_, the probability of choosing *t *is greater than that of *t*'.

3.2. Otherwise (a deletion operation), for all *l *≤ *t *≤ *t*' ≤ *u *and *t *≠ *t*' ≠ *t*_1_, the probability of choosing *t *is less than that of *t*'

We do not choose *t*_2 _(*t*_2 _≠ *t*_1_) uniformly at random in [*l*, *u*], instead, we tend to place addition operations in the front part of p, and deletion operations in the later part of *p*. This is because adding base pairs early and deleting them late during the folding may help stabilize the intermediate secondary structures. (Please see Additional file 1 for the detailed description of the discrete probability.)

$M_{2}$: Let $M_{2}^{t_{1},t_{2}}\left(p\right)$ denote the sequence of actions obtained by swapping $a_{t_{1}}$ with $a_{t_{2}}$. If the resulting sequence of actions is a valid action chain, let it be *q*; otherwise, restart the process. For example, in Figure 1, $M_{2}^{1,8}\left(p\right)$ is not a valid action chain, while $M_{2}^{2,4}\left(p\right)=a_{1},a_{4},a_{3},a_{2},a_{5},\ldots ,a_{8}$ is. *t*_1 _and *t*_2 _are chosen uniformly at random from {(*t*_1_, *t*_2_): 1 ≤ *t*_1 _<*t*_2 _≤ *m*}.

Mutation strategies of type 1 provide methods for shuffling the order of actions of an input pathway and generating slightly different new pathways. However, these strategies are not capable of introducing additional (indirect) base pairs, and the offsprings of a direct pathway produced through type 1 strategies are also direct. In the following, we will describe mutation strategies that are able to construct indirect pathways from a direct pathway.

#### Type 2: introducing indirect pathways by adding a pair of complementary actions

Morgan and Higgs [16] pointed out that the optimal folding paths are generally indirect pathways. This idea was further described by Dotu *et al*. [28]. The temporary formation of base pairs, especially those base pairs that do not belong to *A *∪ *B*, may lower the energies of intermediate structures and thus render better folding pathways. Similarly, temporary deletion and reformation of a base pair also can create an indirect pathway.

$M_{3}$: Let $M_{3}^{t_{1},t_{2},+\left(i,j\right)}\left(p\right)$ denote the sequence of actions obtained by introducing an addition action add*_i,j _*after $a_{t_{1}}$ and its complementary action del*_i,j _*after $a_{t_{2}}$. Let $M_{3}^{t_{1},t_{2}-\left(i,j\right)}\left(p\right)$ denote the sequence of actions obtained by introducing a deletion action del*_i,j _*after $a_{t_{1}}$ and its complementary action add*_i,j _*after $a_{t_{2}}$. For example, in Figure 1, $M_{3}^{1,7,+\left(1,16\right)}\left(p\right)=a_{1},$add_1,16_, a_2_, ..., a_7_, del_1,16_, a_8_. The procedures for computing $M_{3}^{t_{1},t_{2},+\left(i,j\right)}\left(p\right)$ and $M_{3}^{t_{1},t_{2},-\left(i,j\right)}\left(p\right)$ are similar to each other. In the following, we only describe the procedure for computing $M_{3}^{t_{1},t_{2},+\left(i,j\right)}\left(p\right)$.

1. Choose *t*_1 _uniformly at random from the interval [1, *m*], and obtain the associated intermediate structure $S_{t_{1}}$.

2. Find a set of base pairs that neither conflict with nor clash with $S_{t_{1}}$ and choose a base pair (*i*, *j*) uniformly at random from the set.

3. Compute the interval [*l*, *u*], (*t*_1 _<*l *<*u *<*m*), where *l *is the minimum and *u *is the maximum such that for all values *t*_2 _∈ [*l*, *u*] the resulting sequence of actions of $M_{3}^{t_{1},t_{2},+\left(i,j\right)}\left(p\right)$ is a valid action chain.

4. Choose *t*_2 _from the interval [*l*, *u*] with the probability of choosing *t *greater than that of *t*' for all *t *>*t*'. (This is because (*i*, *j*) is not likely to be deleted soon after its formation.)

Mutation strategy $M_{3}$is capable of producing an indirect pathway from a direct pathway. In addition, a proper combination of multiple applications of $M_{3}$ may result in a pathway which simulates the successive formation and deletion of a temporary stack during the folding. Take the pathway *p *in Figure 1 as an example, we can construct a pathway *q *that forms a temporary stack consisting of all the GU base pairs via a multiple application of $M_{3},\;q=M_{3}^{5,7,+\left(3,14\right)}\left(M_{3}^{3,7,+\left(2,15\right)}\left(M_{3}^{1,7,+\left(1,16\right)}\left(p\right)\right)\right)$.

#### Type 3: formation of a single stack or simultaneous formation and deletion of a pair of incompatible stacks

In this section, we will introduce mutation strategies for producing pathways that involve with formation and deletion of stacks. To perform this type of strategies, we first need to find all possible stacks in an RNA sequence *x*. We use the algorithm of Bafna *et al*. [38] to find the set of all possible stacks with more than 3 consecutive base pairs, and denote it by *STA*(*x*). There are two strategies in Type 3: formation of a single stack $\left(M_{4}\right)$ and simultaneous formation and destruction of a pair of incompatible stacks $\left(M_{5}\right)$.

$M_{4}$: Let $M_{4}^{t,h}\left(p\right)$ denote the sequence of actions obtained by forcing the formation of a stack *stack_h _*∈ *STA *after action *a_t_*, where *stack_h _*is compatible with *S_t_*. The following describes the procedure for computing $M_{4}^{t,h}\left(p\right)$.

1. Choose *t *uniformly at random from the interval [1, *m*], and obtain the associated intermediate structure *S_t_*.

2. Find a set of stacks that neither conflict with nor clash with *S_t_*, and pick up a stack *stack_h _*uniformly at random from the set.

3. Ensure that each base pair (*i*, *j*) in {*stack_h _*- *S_t_*} is sequentially (from the innermost base pair to the outmost base pair) formed after *a_t_*.

3.1. If an action add*_i,j _*appears in {*a*_t+1_, . . . , *a_m_*}, move it up and place it after *a_t _*using strategy $M_{1}$.

3.2. Otherwise, introduce a pair of complementary actions add*_i,j _*and del*_i,j _*to *p *after *a_t _*using strategy $M_{3}$.

We can introduce additional stacks that are compatible with *S_t _*using $M_{4}$ by forcing a sequence of addition actions successively forming base pairs in {*stack_h _*- *S_t_*}, after *a_t_*.

**Figure 4:**
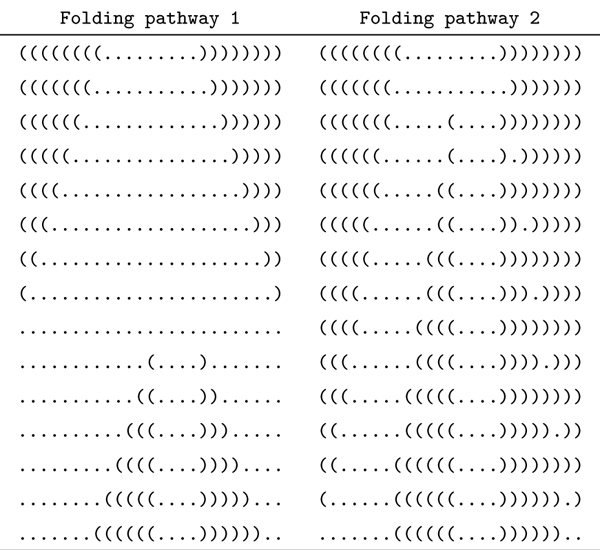
**Two different folding pathways with the identical initial and final secondary structures.** Two different folding pathways with the identical initial and final secondary structures. Left: Folding pathway 1 destroys a stack completely before an incompatible stack is formed. Right: Folding pathway 2 destructs a stack and forms an incompatible stack simultaneously.

$M_{5}$: Let $M_{5}^{t,h}\left(p\right)$ denote the sequence of actions obtained by forcing the formation of a stack *stack_h _*∈ *STA *which is incompatible with *S_t_*, after action *a_t_*. Shown on the right side of Figure 4 is a folding pathway which simultaneously destructs and forms a pair of incompatible stacks. Shown on the left side is a simple folding pathway which has exactly the same start and end structures, while it folds into a single stranded structure during the folding. Usually, the pathway on the right has lower energy barrier than the one on the left because it never folds into a single stranded structure. The folding pathway on the right side of Figure 4 can be introduced using strategy $M_{5}$. And, the procedure for computing $M_{5}^{t,h}\left(p\right)$ is as follows:

1. Choose an arbitrary deletion action *a_t _*= del*_i,j _*from *p*, and obtain the associated intermediate structure *S_t_*.

2. Find a set of stacks which either conflicts with or clashes with *S_t_*, and choose a stack *stack_h _*uniformly at random from the set.

3. For each base pair (*i*', *j*') in {*stack_h _*- *S_t_*} that is compatible with *S_t_*, place add*_i_*_',_*_j_*_' _to *p *after *a_t _*using strategy $M_{4}$.

4. For each base pair (*i*', *j*') in {*stack_h _*- *S_t_*} that is incompatible with *S_t_*,

4.1. Find all the base pairs (i*, j*) in *S_t _*that are incompatible with (*i*', *j*'), and ensure that each base pair (i*, j*) is deleted before the action add*_i_*_',_*_j'_*.

4.2. If a action $\text{de}{\text{l}}_{i^{*},j^{*}}$ appears in {*a_t_*_+1_, . . . , *a_m_*}, move it up before add*_i_*_'_,*_j_*_'_using strategy $M_{1}$.

4.3. Otherwise, introduce a pair of complementary actions $\text{de}{\text{l}}_{i^{*},j^{*}}$ and $\text{ad}{\text{d}}_{i^{*},j^{*}}$using strategy $M_{3}$. 

Using $M_{5}$, we can introduce the simultaneous formation of a stack *stack_h_*, which is incompatible with *S_t_*, and destruction of existent stacks (or base pairs) that hamper the formation of *stack_h_*. Since cooperative formation and destruction of stacks may contribute additional stacking energies for stabilizing the intermediate structures, better folding pathways with lower energy barriers may be rendered.

## Results and discussion

### Benchmark tests

We benchmarked RNAEAPath against existing methods (BARRIEERS [20,21], PathwayHunter [17], Find-path [24], and RNATabuPath [28]) by predicting low energy barrier folding pathways between two designated RNA secondary structures of 18 conformational switches. All the conformational switches were taken from the work of Dotu *et. al *[28]. Five of them are riboswitches, including rb1, rb2, rb3, rb4, and rb5. The metastable structures of these riboswitches have been experimentally determined by inline probing [9,39]. The thirteen remaining cases concern conformational switches, including hok, SL (Spliced leader RNA), s15, s-box leader, thiM leader, ms2, HDV, dsrA, ribD leader, amv, alpha operon and HIV-1 leader. Sequences of these conformational switches can also be obtained from paRNAss web site [40], and some of the metastable secondary structures were computationally determined using RNAbor [41].

We summarize the results computed by PathwayHunter, the results computed by BARRIERS, the results computed by Findpath (with the look ahead parameter *k *= 10), the best results over 1000 runs found by RNATabuPath, and the best results over 1 run and 5 runs found by RNAEAPath in Table 1 respectively. And we use '-' to mark test cases that methods fail to apply to in the table. For all methods, free energies of the intermediate structures of the folding pathways (including PathwayHunter) are evaluated based on the Turner model using RNAeval (with -d1 option) from the Vienna RNA Package [25]. The default configuration parameters of RNAEAPath are as follows. *MAX *is 10, *γ *is 5, L is 100, *ℓ*_1 _is 10, *ℓ*_2 _is 5 and *ℓ*_3 _is 100. Due to the stochastic nature of the evolutionary algorithm, we report the best energy barrier of RNAEAPath found over both 1 run and 5 runs.

**Table 1:** Benchmarks of BARRIERS, PathwayHunter, Findpath, RNATabuPath, and RNAEAPath for predicting folding pathways between conformational switches on the **18 **test cases

Instance	BARRIERS	PathwayHunter	FindPath	RNATabuPath	RNAEAPath	
					
				(*n *= 1000)	(*n *= 1)	(*n *= 5)
rb1	-	-	24.04	24.04	23.2	**22**
rb2	-	10	8.2	7.25	**6.5**	**6.5**
rb3	-	-	22.4	17.9	17.5	**16.7**
rb4	-	-	**16.9**	**16.9**	**16.9**	**16.9**
rb5	-	-	24.54	24.54	**21.44**	**21.44**
hok	-	-	28.5	29.66	20.7	**20.1**
SL	11.80	-	13	**12.9**	13.0	**12.9**
attenuator	8.3	-	8.7	8.6	8.7	**8.5**
s15	6.60	-	7.1	**6.6**	7.1	7.1
sbox leader	-	7.9	**5.2**	**5.2**	**5.2**	**5.2**
thiM leader	-	-	16.13	14.84	**12.3**	**12.3**
ms2	-	11.6	**6.6**	**6.6**	**6.6**	**6.6**
HDV	-	23.53	17.4	17.0	**16.8**	**16.8**
dsrA	8.0	-	8.3	8.2	**8.0**	**8.0**
ribD leader	-	-	10.71	**9.5**	**9.5**	**9.5**
amv	-	12.2	5.8	5.8	**5.74**	**5.74**
alpha operon	-	11.8	6.5	6.5	**6.1**	**6.1**
HIV-1 leader	-	14.3	9.3	11.3	**8.9**	**8.9**

Benchmarks of BARRIERS, PathwayHunter, Findpath, RNATabuPath, and RNAEAPath for predicting folding pathways between conformational switches. Energy barriers (measured in kcal/mol) of the best folding pathways over *n *runs are shown. Boldface numbers are the best energy barriers found by the *heuristic *algorithms.

BARRIERS is the only exact solution that produces indirect pathways based on the Turner model. BARRIERS has already been compared with existing heuristic algorithms on the same test cases in the work of Dotu *et al*. [28]. We put the results of BARRIERS in the table just for the sake of comparison. It has been pointed out that BARRIERS gives provably globally optimal pathways in 4 out of 18 cases (i.e. SL, attenuator, s15 and dsrA). BARRIERS can not be directly applied to 5 cases because either the initial or the end structure is not locally optimal (i.e. rb2, sbox leader, ms2, amv and alpha operon), and can not converge in the remaining cases. Possibly due to the fact that both the number of RNA secondary conformations to consider and the computational resources required increase exponentially with the growing length of the RNA sequence and the growing range of energy barrier. PathwayHunter is an exact algorithm capable of producing the optimal direct folding pathways based on the Nussinov model. PathwayHunter can not be directly applied to 10 cases, because it requires the pair of input structures being able to form a 'pairwise-optimal' bipartite conflicting graph (see the work of Thachuk *et al*. [17] for details). It is not surprising that the performance of the exact algorithm, PathwayHunter, evaluated by free energy (in kcal/mol), is worse than the heuristic algorithms. This is because PathwayHunter is optimized based on the Nussinov model and only produces direct pathways, while the optimal direct pathways predicted based on the Nussinov model may not be the optimal pathways (considering both direct and indirect pathways) based on the Turner model. All the remaining three methods are heuristics capable of producing both direct and indirect pathways based on the Turner model. Findpath produces folding pathways very quickly, however it performs worse than both RNATabuPath and RNAEAPath in most cases. RNATabuPath performs better than Findpath, but produces less optimal pathways than RNAEAPath. The energy barriers predicted by RNAEAPath over 5 runs are exactly the same as RNATabuPath in 5 cases, worse in 1 case, and better in all the remaining 12 cases.

Other heuristic algorithms (including a greedy algorithm of Voss *et al*. [26], a semi-greedy modification of the greedy algorithm, a greedy algorithm of Morgan, and Higgs [16] for predicting direct pathways and a variant of the Morgan-Higgs greedy algorithm capable of producing indirect pathways), that have been shown to perform considerably worse than RNATabuPath [28], are not listed.

**Figure 5:**
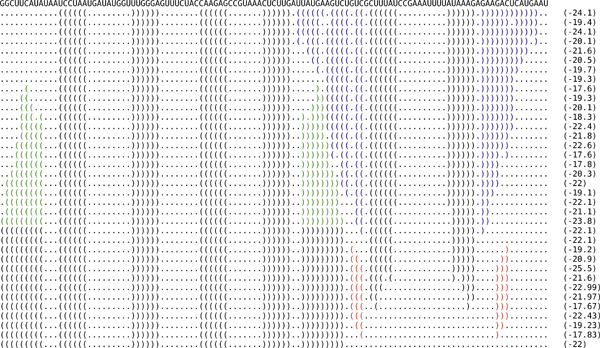
**The best folding pathway predicted for rb2.** The near optimal indirect pathway between the two conformational secondary structures of the adenine riboswitch from *V*. *vulnificus *(rb2) predicted RNAEAPath.

By analyzing the best folding pathways produced by RNAEAPath, we found that most high-quality pathways involve the melting of stacks in the initial structure, the (possibly simultaneous) construction of stacks in the final structure, and the formation of auxiliary temporary stacks for obtaining folding pathways with lower energy barriers. We may take the lowest energy barrier folding pathway of rb2 found by RNAEAPath, shown in Figure 5 as an example. The stack colored in red is an auxiliary temporary stack introducing intermediate structures with lower free energies (which is constructed using $M_{4}$). Some of the stacks in the initial structure (in blue) are gradually melting, while at the same time, an incompatible stack (in green) is being formed (which is constructed using $M_{5}$). The stack colored in red is an auxiliary temporary stack introducing intermediate structures with lower free energies. This example convinces us that the advantages of RNAEAPath mainly come from employing mutation strategies that guide the construction of folding pathways by the formation and destruction of stacks and introducing additional stacking interactions that are important for stabilizing the intermediate structures. Detailed low energy barrier folding pathways for all the test cases are available on RNAEAPath web site.

### Control parameters and performance

In order to evaluate the performance of RNAEAPath with different parameter configurations, we played with several other control parameters, including *ℓ*_1_, the number of top offsprings preserved in the next generation, varying from 1 to 16, *ℓ*_3_, the size of population in each generation, varying from 80 to 120 and L, the total number of offsprings each individual is expected to produce, varying from 80 to 120. The detailed results are shown in Additional file 1. In general, RNAEAPath produces pathways of roughly the same quality for most test cases with different control parameters, among which the default parameter setting is the best.

**Figure 6:**
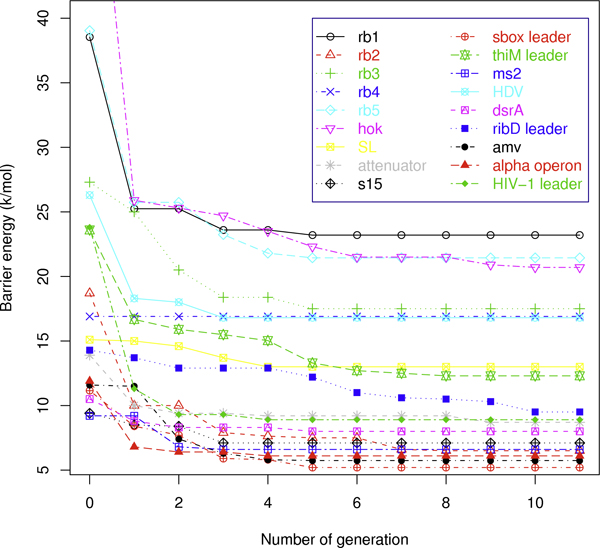
**Performance of RNAEAPath in each generation.** Energy barriers (in kcal/mol) of the best folding pathways of 18 conformational switches by the end of each generation during a typical run of RNAEAPath using the default parameters.

We explored the relationship between the performance of RNAEAPath and the number of generations completed by plotting energy barriers of the best folding pathways produced by RNAEAPath with the default parameters in each generation, as shown in Figure 6. In general, the energy barriers decrease dramatically in the first one or two generations, and then the decrements slow down and finally plateau within 10 generations. For instance, in the case of rb3, the predicted energy barriers of folding pathways in the initial population is 27.3 kcal/mol. It decreases by 7.2 kcal/mol (24.9%) through the first two generations and decreases by 2.5 kcal/mol (9.2%) through the next three generations. Through all the remaining generations, no further improvement is made.

We also evaluated the execution time for each run of RNAEAPath. All the tests were performed on a 32 bit PC with 2.4 GHz Quad-processor and 3.2 GB memory, running Fedora 11. With the default control parameters, RNAEAPath terminates in 1 minute in the best case (rb4), 445 minutes in the worst case (hok), and 43 minutes on average. The detailed running times are shown in Additional file 1. We did not perform direct comparisons between the running time of RNATabuPath and that of RNAEAPath, since RNATabuPath is only accessible via web server.

## Conclusions

In conclusion, we have presented a new algorithm, RNAEAPath, for predicting low energy barrier folding pathways between conformational structures. RNAEAPath guides the construction of folding pathways through the destruction and formation of RNA stacks using various types of mutation strategies, and integrates them in a well-established computational framework of evolutionary algorithm. These mutation strategies can help reduce the search space and make it easier to jump out of local optima. By analyzing the results, we confirmed that most of the best folding pathways involve the formation of auxiliary stacks, or involve the cooperative formation and disruption of incompatible stacks. The benchmarking results show that RNAEAPath outperforms the existing heuristics on most test cases. We believe that this is because the construction of folding pathways in RNAEAPath captures important biological findings.

## Competing interests

The authors declare that they have no competing interests.

## Authors' contributions

SZ and YL conceived and designed the project. YL implemented the program and ran benchmark tests for the paper. SZ and YL both drafted the manuscript and approved the final manuscript.

## Supplementary Material

Additional file 1 **Supplementary data for predicting folding pathways between RNA conformational structures guided by RNA stacks.** Supplementary data for predicting folding pathways between RNA conformational structures guided by RNA stacks in a PDF file. Click here for file
