# Supplementary material for: Predicting folding pathways between RNA conformational structures guided by RNA stacks
Source: BMC Bioinformatics. 2012 Mar 21;13(Suppl 3):S5. doi: 10.1186/1471-2105-13-S3-S5 (PMC3402921; doi:10.1186/1471-2105-13-S3-S5)
Supplement: Additional file 1 — Supplementary data for predicting folding pathways between RNA conformational structures guided by RNA stacks. Supplementary data for predicting folding pathways between RNA conformational structures guided by RNA stacks in a PDF file. [file 1471-2105-13-S3-S5-S1.pdf]

Supplementary Data

Yuan Li and Shaojie Zhang
Department of Electrical Engineering and Computer Science
University of Central Florida
Orlando, FL, USA, 32816-2362

1 The Discrete Probability Distribution

We define a discrete probability distribution (similar to the discrete Gaussian distribution) over a sample space. Let X be a random variable over $\mathbb{R}$ following a normal distribution with mean μ ($\mu = 0$) and variance σ^2. Consider a sample space of n distinguishable objects $\mathcal{V} = \{v_1, v_2, \ldots, v_n\}$. The $\mathcal{V}$-*distribution* selects a sample v with probability $Pr(v)$ ($\Pr(v = v_i) = Pr((i-1)/n \leq |X| \leq i/n)$ for $1 \leq i \leq n-1$ and $Pr(v = v_n) = Pr(|X| \geq (n-1)/n)$). The default value of σ^2 is $1/12$, so that $Pr(|X| \geq 1) = 0.0005$. Consider the set $\{p_l, \ldots, p_{(l+n-1)}\}$ and construct $\mathcal{V}$ as follows. If a_t is an addition operation, then $\mathcal{V} = \{v_1 = p_l, \ldots, v_{(l+n-1)} = p_u\}$. If a_t is a deletion operation, then $\mathcal{V} = \{v_1 = p_{(l+n-1)}, \ldots, v_n = p_l\}$. The actions chain q is chosen from $\mathcal{V}$ with the $\mathcal{V}$-distribution.

2 Performance of RNAEAPath using different control parameters.

The performance of RNAEAPath using different control parameters is evaluated. Table 1 shows the energy barriers of the best folding pathways found by RNAEAPath over 5 runs with the number of the preserved top offsprings ℓ_1 ($\ell_1$varies from 1 to 16). Table 2 shows the performance of RNAEAPath over 5 runs with the expected size of population ℓ_3 (ℓ_3 varies from 80 to 120). Table 3 shows the performance of RNAEAPath over 5 runs with the number of offsprings $\mathfrak{L}$ (for ach individual $\mathfrak{L}$ varies from 80 to 120).

Table 1: Energy barriers of the best folding pathways found by RNAEAPath over 5 runs with different control parameters: ℓ_1, the number of top offsprings preserved in the next generation, varies from 1 to 16. The energies are measured in kcal/mol.

Instance	Control Parameter: ℓ_1					
	1	4	7	10	13	16
rb1	**22**	**22**	**22**	**22**	**22**	**22**
rb2	7.4	7.5	**10**	**6.5**	**6.5**	**6.5**
rb3	**16.7**	**16.7**	17.1	**16.7**	**16.7**	**16.7**
rb4	**16.9**	**16.9**	**16.9**	**16.9**	**16.9**	**16.9**
rb5	**21.44**	**21.44**	**21.44**	**21.44**	**21.44**	**21.44**
hok	20.2	**20.1**	20.2	**20.1**	**20.1**	**20.1**
SL	13	13	**12.9**	**12.9**	13	13
attenuator	8.6	**8.5**	**8.5**	**8.5**	**8.5**	**8.5**
s15	**6.6**	7.1	7.1	7.1	**6.6**	7.1
sbox leader	**5.2**	**5.2**	**5.2**	**5.2**	**5.2**	**5.2**
thiM leader	**12.3**	**12.3**	**12.3**	**12.3**	**12.3**	**12.3**
ms2	**6.6**	**6.6**	**6.6**	**6.6**	**6.6**	**6.6**
HDV	**16.7**	16.8	**16.7**	16.8	16.8	16.8
dsrA	**8**	**8**	**8**	**8**	**8**	**8**
ribD leader	**9.5**	**9.5**	**9.5**	**9.5**	**9.5**	**9.5**
amv	**5.74**	**5.74**	**5.74**	**5.74**	**5.74**	**5.74**
alpha operon	6.5	**6.1**	**6.1**	**6.1**	**6.1**	**6.1**
HIV-1 leader	**8.9**	**8.9**	**8.9**	**8.9**	**8.9**	**8.9**

Table 2: Energy barriers of the best folding pathways found by RNAEAPath over 5 runs with different control parameter: ℓ_3, the size of population in each generation varies from 80 to 120. The energies are measured in kcal/mol.

Instance	Control Parameter: ℓ_3				
	80	90	100	110	120
rb1	**22**	**22**	**22**	**22**	22.4
rb2	**6.5**	7.4	**6.5**	**6.5**	**6.5**
rb3	**16.7**	17.1	**16.7**	**16.7**	**16.7**
rb4	**16.9**	**16.9**	**16.9**	**16.9**	**16.9**
rb5	**21.44**	**21.44**	**21.44**	**21.44**	**21.44**
hok	**20.1**	20.9	**20.1**	20.7	**20.1**
SL	13	13	**12.9**	13	13
attenuator	**8.5**	8.6	**8.5**	8.6	**8.5**
s15	7.1	**6.6**	7.1	**6.6**	**6.6**
sbox leader	**5.2**	**5.2**	**5.2**	**5.2**	**5.2**
thiM leader	12.3	12.3	12.3	**12**	**12**
ms2	**6.6**	**6.6**	**6.6**	**6.6**	**6.6**
HDV	16.8	16.8	16.8	**16.7**	16.8
dsrA	**8**	**8**	**8**	**8**	**8**
ribD leader	**9.5**	**9.5**	**9.5**	**9.5**	**9.5**
amv	**5.74**	**5.74**	**5.74**	**5.74**	**5.74**
alpha operon	**6.1**	**6.1**	**6.1**	**6.1**	**6.1**
HIV-1 leader	**8.9**	**8.9**	**8.9**	**8.9**	**8.9**

Table 3: Energy barriers of the best folding pathways found by RNAEAPath over 5 runs with different control parameters: $\mathfrak{L}$, the number of offsprings that each individual should generate varies from 80 to 120. The energies are measured in kcal/mol.

Instance	Control Parameter: $\mathfrak{L}$				
	80	90	100	110	120
rb1	**22**	**22**	**22**	**22**	**22**
rb2	7.4	**6.5**	**6.5**	**6.5**	**6.5**
rb3	17.5	**16.7**	**16.7**	**16.7**	**16.7**
rb4	**16.9**	**16.9**	**16.9**	**16.9**	**16.9**
rb5	**21.44**	**21.44**	**21.44**	**21.44**	**21.44**
hok	20.5	**20.1**	**20.1**	**20.1**	**20.1**
SL	**12.9**	13	**12.9**	13	13
attenuator	**8.5**	**8.5**	**8.5**	8.6	**8.5**
s15	7.1	**6.6**	7.1	7.1	7.1
sbox leader	**5.2**	**5.2**	**5.2**	**5.2**	**5.2**
thiM leader	12.3	12.3	12.3	**12**	12.1
ms2	**6.6**	**6.6**	**6.6**	**6.6**	**6.6**
HDV	**16.7**	16.8	16.8	**16.7**	16.8
dsrA	**8**	**8**	**8**	**8**	**8**
ribD leader	**9.5**	**9.5**	**9.5**	**9.5**	**9.5**
amv	**5.74**	**5.74**	**5.74**	**5.74**	**5.74**
alpha operon	**6.1**	**6.1**	**6.1**	**6.1**	**6.1**
HIV-1 leader	**8.9**	**8.9**	**8.9**	**8.9**	**8.9**

3 Running Time

Table 4 shows the running time of RNAEAPath on 18 conformational switches using the default parameters. The tests are performed on a 32 bit, 2.4 GHz Quad-processor, 3.2GB memory computer, the operating system is Fedora 11. The minimum execution time is 1 minute (in the case of rb4) and 421 minutes (in the case of hok), and on average RNAEAPath terminates in 43 minutes.

Table 4: Running time of RNAEAPath on 18 conformational switches using the default parameters.

Instance	Running Time (minutes)
rb1	34
rb2	16
rb3	22
rb4	1
rb5	17
hok	421
SL	13
attenuator	13
s15	10
sbox leader	20
thim leader	45
ms2	10
HDV	20
dsrA	13
ribD leader	52
amv	14
alpha operon	15
HIV-1 leader	34

4 The Best Folding Pathways Found by RNAEAPath

The best folding pathways between two conformational structures of 18 riboswitches found by RNAEAPath over 5 runs using the default parameters are shown as follows.

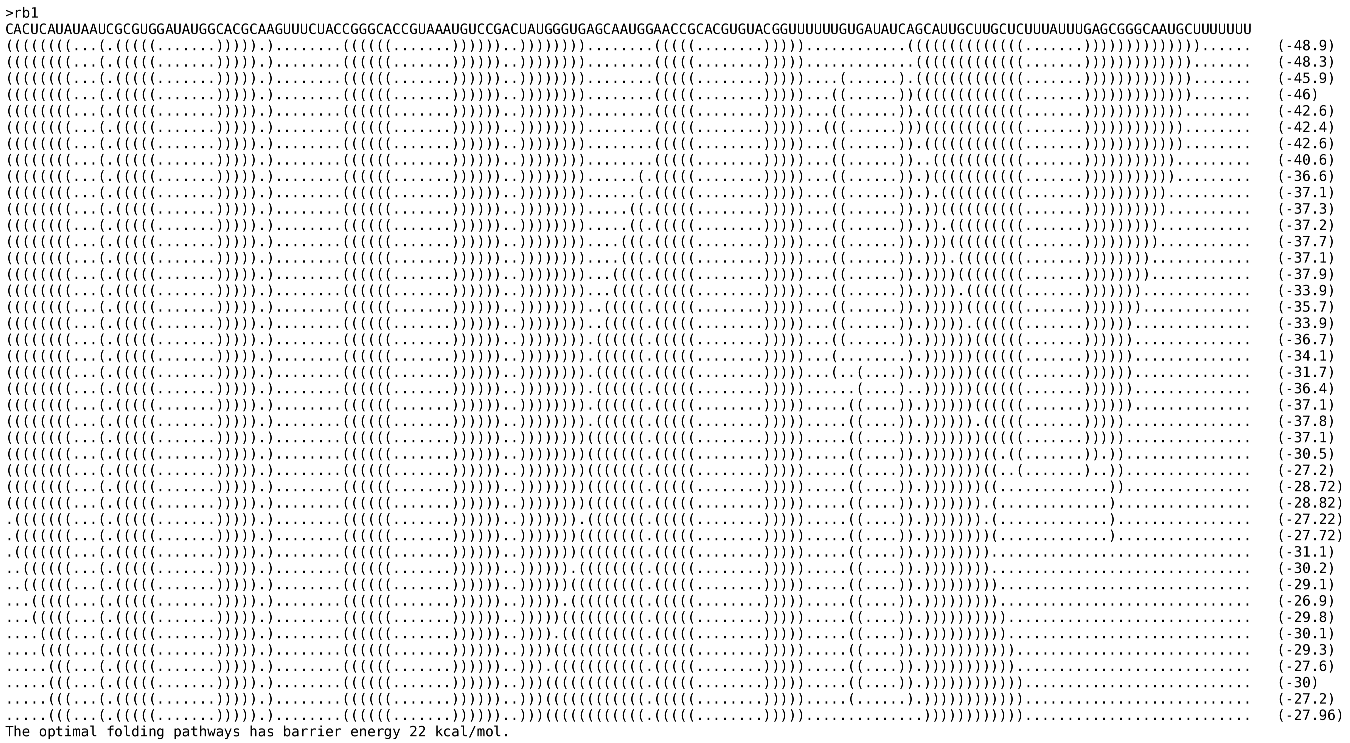

Figure 1: The best folding pathway between two conformational structures of rb1 found by RNAEAPath over 5 runs using the default parameters.

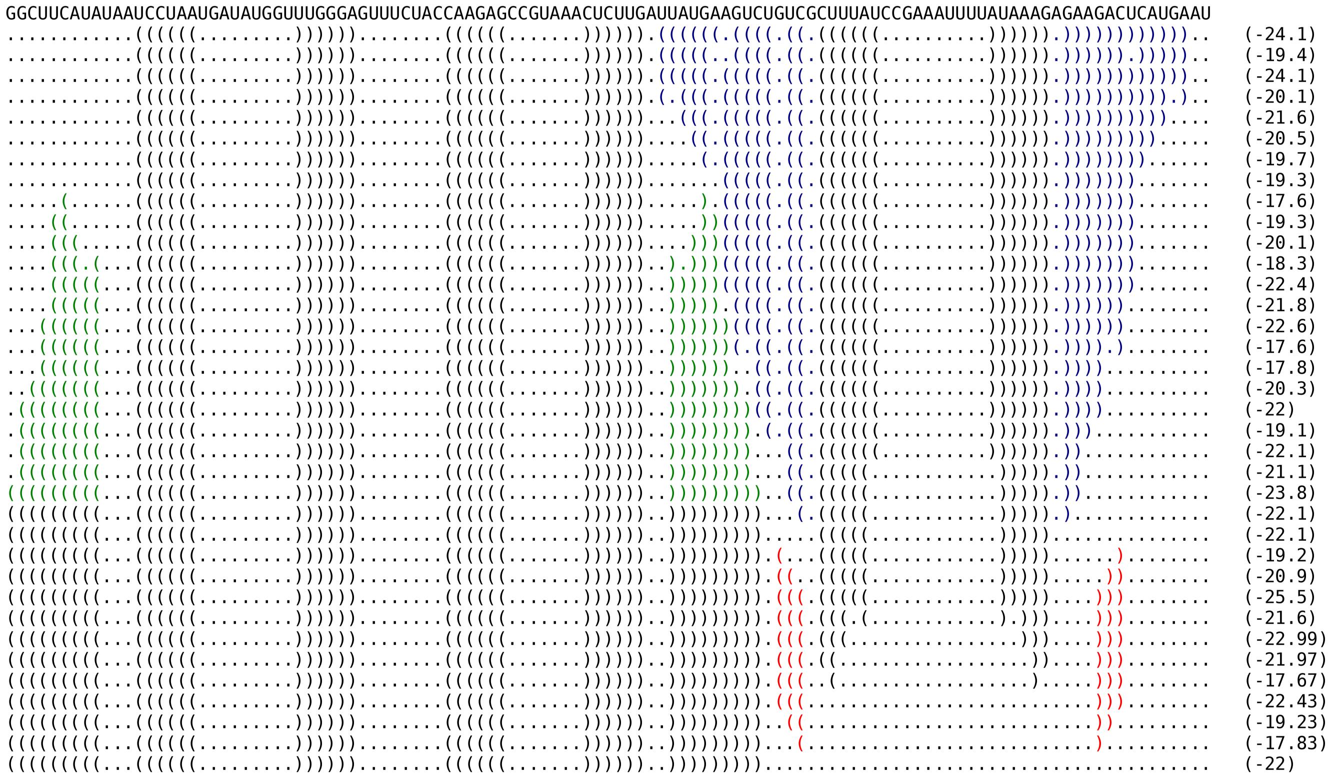

Figure 2: The best folding pathway between two conformational structures of rb2 found by RNAEAPath over 5 runs using the default parameters.

```
>rb3
CUCUUAUCAAGAGAGGUGGAGGGACUGGCCCGAUGAAACCCGGCAACCAGCCUUAGGGCAUGGUGCCAAUUCCUGCAGCGGUUUCGCGUUGAAAGAUGAGAGAUUCUUGUAGUCUCUUCUUUUAGCGAAGGGACUUUUUUU
((((((((....(.(((...(((.....)))......))))(((..((((((....))).))).)))........(((((......)))))...))))))))........((((((((.......))))))))........   (-41.9)
((((((((......(((...(((.....)))......))).(((..((((((....))).))).)))........(((((......)))))...))))))))........((((((((.......))))))))........   (-43.2)
.(((((((......(((...(((.....)))......))).(((..((((((....))).))).)))........(((((......)))))...))))))).........((((((((.......))))))))........   (-40.4)
..((((((......(((...(((.....)))......))).(((..((((((....))).))).)))........(((((......)))))...)))))).........((((((((.......))))))))........   (-38.8)
.(((((((......(((...(((.....)))......))).(((..((((((....))).))).)))........(((((......)))))...))))))).........((((((((.......))))))))........   (-40.4)
.(((((((......(((...(((.....)))......))).(((..((((((....))).))).)))........(((((......)))))...)))))))..........(((((((.......)))))))........   (-37)
((((((((......(((...(((.....)))......))).(((..((((((....))).))).)))........(((((......)))))...))))))))..........(((((((.......)))))))........   (-39.8)
((.(((((......(((...(((.....)))......))).(((..((((((....))).))).)))........(((((......)))))...))))).))..........(((((((.......)))))))........   (-33.6)
((((((((......(((...(((.....)))......))).(((..((((((....))).))).)))........(((((......)))))...))))))))..........(((((((.......)))))))........   (-39.8)
((((((((......(((...(((.....)))......))).((((.((((((....))).)))))))........(((((......)))))...))))))))..........(((((((.......)))))))........   (-44.3)
(((.((((......(((...(((.....)))......))).((((.((((((....))).)))))))........(((((......)))))...)))).)))..........(((((((.......)))))))........   (-39.8)
(((..(((......(((...(((.....)))......))).((((.((((((....))).)))))))........(((((......)))))...)))..)))..........(((((((.......)))))))........   (-37.9)
(((...((......(((...(((.....)))......))).((((.((((((....))).)))))))........(((((......)))))...))...)))..........(((((((.......)))))))........   (-36.1)
(((....(......(((...(((.....)))......))).((((.((((((....))).)))))))........(((((......)))))...)....)))..........(((((((.......)))))))........   (-34.1)
(((...........(((...(((.....)))......))).((((.((((((....))).)))))))........(((((......))))).........)))..........(((((((.......)))))))........   (-35.9)
((((..........(((...(((.....)))......))).((((.((((((....))).)))))))........(((((......)))))........))))..........(((((((.......)))))))........   (-36.6)
(((((.........(((...(((.....)))......))).((((.((((((....))).)))))))........(((((......))))).......)))))..........(((((((.......)))))))........   (-38.3)
(((((.........(((...(((.....)))......))).(((..((((((....))).))).)))........(((((......))))).......)))))..........(((((((.......)))))))........   (-33.8)
(((((.........(((...(((.....)))......))).(((.(((((((....))).)))))))........(((((......))))).......)))))..........(((((((.......)))))))........   (-38.3)
((((((........(((...(((.....)))......))).(((.(((((((....))).)))))))........(((((......)))))......))))))..........(((((((.......)))))))........   (-39.3)
(((((((.......(((...(((.....)))......))).(((.(((((((....))).)))))))........(((((......)))))....)))))))..........(((((((.......)))))))........   (-40)
((((((((......(((...(((.....)))......))).(((.(((((((....))).)))))))........(((((......)))))...))))))))..........(((((((.......)))))))........   (-44.3)
((((((((......(((...(((.....)))......))).(((.(((((((....))).)))))))........(((((......)))))...))))))))...........((((((.......))))))..........   (-42.9)
((((((((......(((...(((.....)))......))).(((.(((((((....))).)))))))........(((((......)))))...))))))))............(((((.......)))))...........   (-40)
((((((((......(((...(((.....)))......))).(((.(((((((....))).)))))))........(((((......)))))...))))))))..............((((.......))))............   (-39.5)
((((((((......(((...(((.....)))......))).(((.(((((((....))).)))))))........(((((......)))))...))))))))...............(((.......)))............   (-36.4)
((((((((......(((...(((.....)))......))).(((.(((((((....))).)))))))........(((((......)))))...))))))))................((.......))..............   (-35.6)
((((((((......(((...(((.....)))......))).(((.(((((((....))).)))))))........(((((......)))))...)))))))).................(.......)...............   (-33.9)
((((((((......(((...(((.....)))......))).(((.(((((((....))).)))))))........(((((......)))))...))))))))...........................................   (-37.2)
.(((((((......(((...(((.....)))......))).(((.(((((((....))).)))))))........(((((......)))))...)))))))............................................   (-34.4)
..((((((......(((...(((.....)))......))).(((.(((((((....))).)))))))........(((((......)))))...))))))..............................................   (-32.8)
...(((((......(((...(((.....)))......))).(((.(((((((....))).)))))))........(((((......)))))...)))))...............................................   (-29.9)
...(((((......(((...(((.....)))......))).(((.(((((((....))).)))))))........(((((......)))))...)))))(............)...........................   (-25.62)
...(((((......(((...(((.....)))......))).(((.(((((((....))).)))))))........(((((......)))))...)))))((..........))...........................   (-26.3)
....((((......(((...(((.....)))......))).(((.(((((((....))).)))))))........(((((......)))))...)))).((..........))...........................   (-25.9)
....((((......(((...(((.....)))......))).(((.(((((((....))).)))))))........(((((......)))))...))))(((..........)))..........................   (-25.6)
.....(((......(((...(((.....)))......))).(((.(((((((....))).)))))))........(((((......)))))...))).(((..........)))..........................   (-25.3)
.....(((......(((...(((.....)))......))).(((.(((((((....))).)))))))........(((((......)))))...)))((((..........))))........................   (-28.6)
......((......(((...(((.....)))......))).(((.(((((((....))).)))))))........(((((......)))))...)).((((..........))))........................   (-28.2)
.......(......(((...(((.....)))......))).(((.(((((((....))).)))))))........(((((......)))))...)..((((..........))))........................   (-26.6)
..............(((...(((.....)))......))).(((.(((((((....))).)))))))........(((((......))))).....((((..........))))........................   (-28.5)
..............(((...(((.....)))......))).(((.(((((((....))).)))))))........(((((......)))))....(.((((..........)))).).......................   (-26.2)
..............(((...(((.....)))......))).(((.(((((((....))).)))))))........(((((......)))))...((.((((..........)))).))......................   (-30)
..............(((...(((.....)))......))).(((.(((((((....))).)))))))........(((((......)))))..(((.((((..........)))).)))....................   (-30.6)
............(.(((...(((.....)))......))))(((.(((((((....))).)))))))........(((((......)))))..(((.((((..........)))).)))....................   (-29.3)
............(.(((...(((.....)))......))))(((.(((((((....))).)))))))........(((((......))))).((((.((((..........)))).))))...................   (-29.9)
............(.(((...(((.....)))......))))(((.(((((((....))).)))))))........(((((......)))))(((((.((((..........)))).)))))..................   (-29.7)
............(.(((...(((.....)))......))))(((..((((((....))).))).)))........(((((......)))))(((((.((((..........)))).)))))..................   (-25.2)
The optimal folding pathways has barrier energy 16.7 kcal/mol.
```

Figure 3: The best folding pathway between two conformational structures of rb3 found by RNAEAPath over 5 runs using the default parameters.

```
>rb4
GACUCGGGGUGCCCUUCUGCGUGAAGGCUGAGAAAUACCCGUAUCACCUGAUCUGGAUAAUGCCAGCGUAGGGAAGUCACGGACCACCAGGUCAUUGCUUCUUCACGUUAUGGCAGGAGCAAACUAUGCAAGUCGACCUGUGGUCC
((((((((.((((.(((.....))))))......)..)))).....(((...((((......))))...)))..))))..((((((.((((((.(((((((((((.....))).))))))))...........))))))))))))   (-42.72)
.(((((((.((((.(((.....))))))......)..)))).....(((...((((......))))...)))..)))...((((((.((((((.(((((((((((.....))).))))))))...........))))))))))))   (-38.62)
..((((((.((((.(((.....))))))......)..)))).....(((...((((......))))...)))..))....((((((.((((((.(((((((((((.....))).))))))))...........))))))))))))   (-37.52)
..((((((..(((.(((.....))))))........)))).....(((...((((......))))...)))..))....((((((.((((((.(((((((((((.....))).))))))))...........))))))))))))   (-43.42)
...(((((..(((.(((.....))))))........)))).....(((...((((......))))...)))..).....((((((.((((((.(((((((((((.....))).))))))))...........))))))))))))   (-40.62)
....((((..(((.(((.....))))))........)))).....(((...((((......))))...))).......((((((.((((((.(((((((((((.....))).))))))))...........))))))))))))   (-45.12)
....((((..(((.(((.....))))))........)))).....(((...((((......))))...))).......((((((.((((((.(((.(((((((.....))).)))).)))...........))))))))))))   (-38.52)
....((((..(((.(((.....))))))........)))).....(((...((((......))))...))).......((((((.((((((.(((..((((((.....))).)))..)))...........))))))))))))   (-36.32)
....((((..(((.(((.....))))))........)))).....(((...((((......))))...))).......((((((.((((((.(((...(((((.....))).))...)))...........))))))))))))   (-34.82)
....((((..(((.(((.....))))))........)))).....(((...((((......))))...))).......((((((.((((((.(((....((((.....))).)....)))...........))))))))))))   (-31.72)
....((((.((((.(((.....))))))......)..)))).....(((...((((......))))...))).......((((((.((((((.(((....((((.....))).)....)))...........))))))))))))   (-25.82)
The optimal folding pathways has barrier energy 16.9 kcal/mol.
```

Figure 4: The best folding pathway between two conformational structures of rb4 found by RNAEAPath over 5 runs using the default parameters.

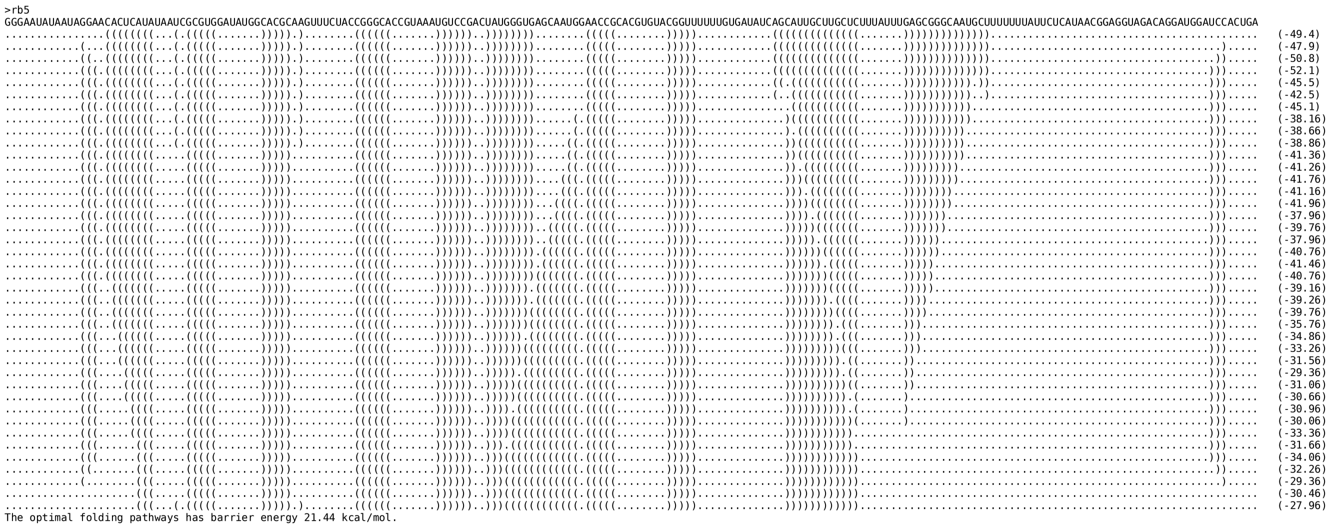

Figure 5: The best folding pathway between two conformational structures of rb5 found by RNAEAPath over 5 runs using the default parameters.

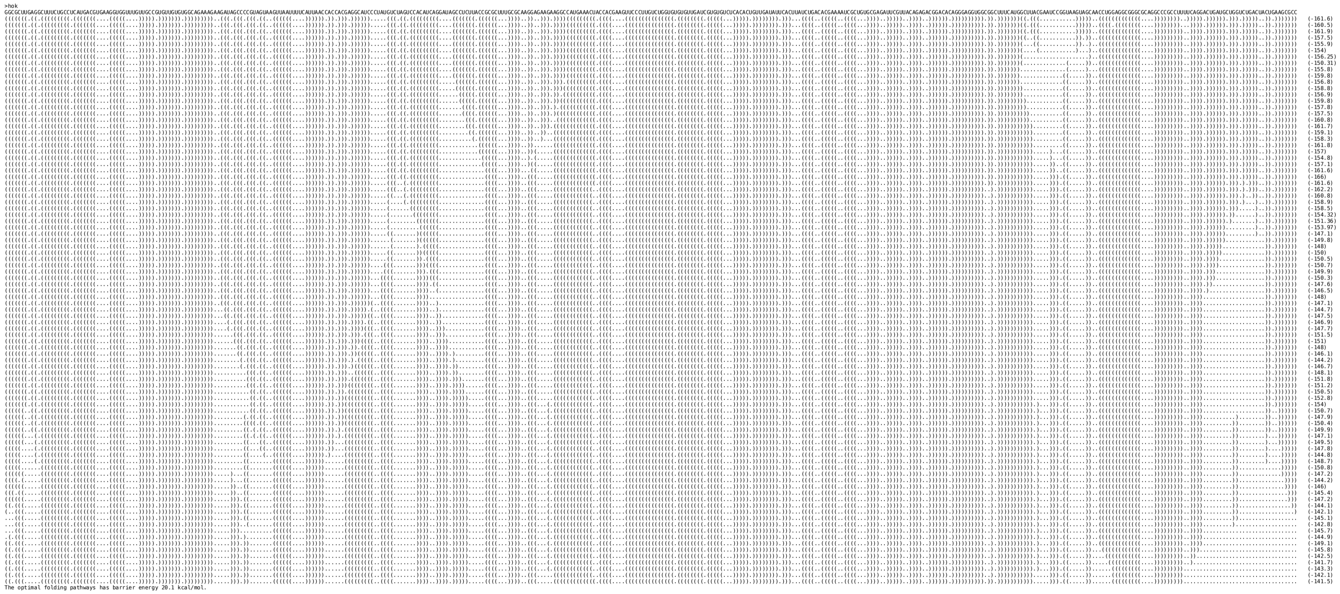

Figure 6: The best folding pathway between two conformational structures of hok found by RNAEAPath over 5 runs using the default parameters.

```
>SplicedLeaderAB
AACUAAAACAAUUUUUGAAGAACAGUUUCUGUACUUCAUUGGUAUGUAGAGACUUC
..((...((((((..(((((.((((...)))).)))))..))).)))..)).....   (-10.7)
..(....((((((..(((((.((((...)))).)))))..))).)))...).....   (-7.8)
.......((((((..(((((.((((...)))).)))))..))).))).........   (-10.1)
.......(((.((..(((((.((((...)))).)))))..))..))).........   (-6.1)
.......(((..(..(((((.((((...)))).)))))..)...))).........   (-7.4)
.......(((.....(((((.((((...)))).)))))......))).........   (-8.1)
.......(((......((((.((((...)))).)))).......))).........   (-5.82)
.......(((.......(((.((((...)))).)))........))).........   (-2.56)
.......(((........((.((((...)))).)).........))).........   (-1.53)
.......(((.........(.((((...)))).)..........))).........   (-0.01)
.......(((...........((((...))))............))).........   (-0.2)
.......(((...........((((...)))).(.......)..))).........   (1.5)
.......(((...........((((...))))((.......)).))).........   (0.7)
.......(((...........((((...))))(((.....))).))).........   (-0.7)
.......(((............(((...))).(((.....))).))).........   (0.2)
.......(((............(((...)))((((.....))))))).........   (0.2)
........((............(((...)))((((.....))))))..........   (1.8)
........(.............(((...)))((((.....)))).)..........   (1.9)
......................(((...)))((((.....))))............   (0.1)
.......................((...)).((((.....))))............   (2.2)
.......................((...))(((((.....)))))...........   (1.4)
........................(...).(((((.....)))))...........   (1.9)
..............................(((((.....)))))...........   (-2.6)
........................(.....(((((.....))))).......)...   (0.8)
........................((....(((((.....)))))......))...   (-0.9)
........................(((...(((((.....))))).....)))...   (-2.4)
........................(((..((((((.....))))))....)))...   (-2.4)
.......................((((..((((((.....))))))....))))..   (-3)
.......................(((((.((((((.....))))))...)))))..   (-3.9)
.......................((((((((((((.....))))))..))))))..   (-7.1)
.......................((((((.(((((.....)))))...))))))..   (-7.3)
.......................((((((((((((.....)))))..)))))))..   (-9)
The optimal folding pathways has barrier energy 12.9.
```

Figure 7: The best folding pathway between two conformational structures of SL found by RNAEAPath over 5 runs using the default parameters.

```
>attenuator
AUCCAGGAGGCUAGCGCGUGAGAAGAGAAACGGAAAACAGCGCCUGAAAGCCUCCCAGUGGAGGCUUUUUUUG
...((((..(((..(.(((..........)))).....))).)))).((((((((....))))))))......   (-21.6)
...((((..(((..(.((............))).....))).)))).((((((((....))))))))......   (-19.7)
...((((..(((..(.(..............)).....))).)))).((((((((....))))))))......   (-17.24)
...((((..(((..(.................).....))).)))).((((((((....))))))))......   (-17.13)
...((((..(((..(.(....)..........).....))).)))).((((((((....))))))))......   (-17.7)
...((((..(((....(....)................))).)))).((((((((....))))))))......   (-19.65)
...((((..(((....(....).........(.....)))).)))).((((((((....))))))))......   (-19.5)
...((((..((.....(....).........(.....).)).)))).((((((((....))))))))......   (-20.1)
...((((..(......(....).........(.....)..).)))).((((((((....))))))))......   (-16.1)
...((((..(....(.(....).........(.....).)).)))).((((((((....))))))))......   (-13.4)
...((((.......(.(....).........(.....).)..)))).((((((((....))))))))......   (-14)
...((((......((.(....).........(.....).)).)))).((((((((....))))))))......   (-18.7)
...(((.......((.(....).........(.....).))..))).((((((((....))))))))......   (-14.1)
...(((...(...((.(....).........(.....).)).)))).((((((((....))))))))......   (-13.1)
...(((...((..((.(....).........(.....).))))))).((((((((....))))))))......   (-15.3)
...(((...((..((.(....).........(.....).)))))))(((((((((....))))))))).....   (-15.1)
....((...((..((.(....).........(.....).)))))).(((((((((....))))))))).....   (-13.2)
....((...((..((.(....).........(.....).))))))((((((((((....))))))))))....   (-13.8)
.....(...((..((.(....).........(.....).))))).((((((((((....))))))))))....   (-13.3)
.........((..((.(....).........(.....).))))..((((((((((....))))))))))....   (-16.1)
........(((..((.(....).........(.....).))))).((((((((((....))))))))))....   (-19.8)
.......((((..((.(....).........(.....).))))))((((((((((....))))))))))....   (-20.4)
The optimal folding pathways has barrier energy 8.5 kcal/mol.
```

Figure 8: The best folding pathway between two conformational structures of attenuator found by RNAEAPath over 5 runs using the default parameters.

```
>s15
CUGGGAUCGCUGAAUUAGAGAUCGGCGUCCUUUCAUUCUAUAUACUUUGGAGUUUUAAAAUGUCUCUAAGUACU
..((((.((((((........))))))))))...........(((((.((((...........)))))))))..   (-17.9)
..((((.((((((........))))))))))...........((((..((((...........)))).))))..   (-14.3)
..((((.((((((........))))))))))...........((((.(((((...........)))))))))..   (-17.9)
..((((.((((((........))))))))))...........(((..(((((...........))))).)))..   (-13.9)
..((((.((((((........))))))))))...........(((.((((((...........)))))))))..   (-17.9)
...(((.((((((........)))))))))............(((.((((((...........)))))))))..   (-17.2)
...((..((((((........)))))).))............(((.((((((...........)))))))))..   (-12.4)
...(...((((((........))))))..)............(((.((((((...........)))))))))..   (-11.5)
.......((((((........))))))...............(((.((((((...........)))))))))..   (-12.6)
..(....((((((........))))))......)........(((.((((((...........)))))))))..   (-10.8)
..(..(.((((((........))))))...)..)........(((.((((((...........)))))))))..   (-14.4)
.((..(.((((((........))))))...)..)).......(((.((((((...........)))))))))..   (-15)
The optimal folding pathways has barrier energy 7.1.
```

Figure 9: The best folding pathway between two conformational structures of s15 found by RNAEAPath over 5 runs using the default parameters.

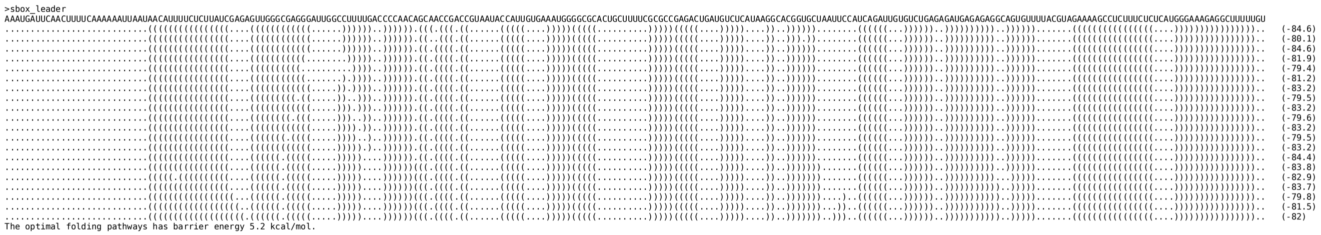

Figure 10: The best folding pathway between two conformational structures of sbox leader found by RNAEAPath over 5 runs using the default parameters.

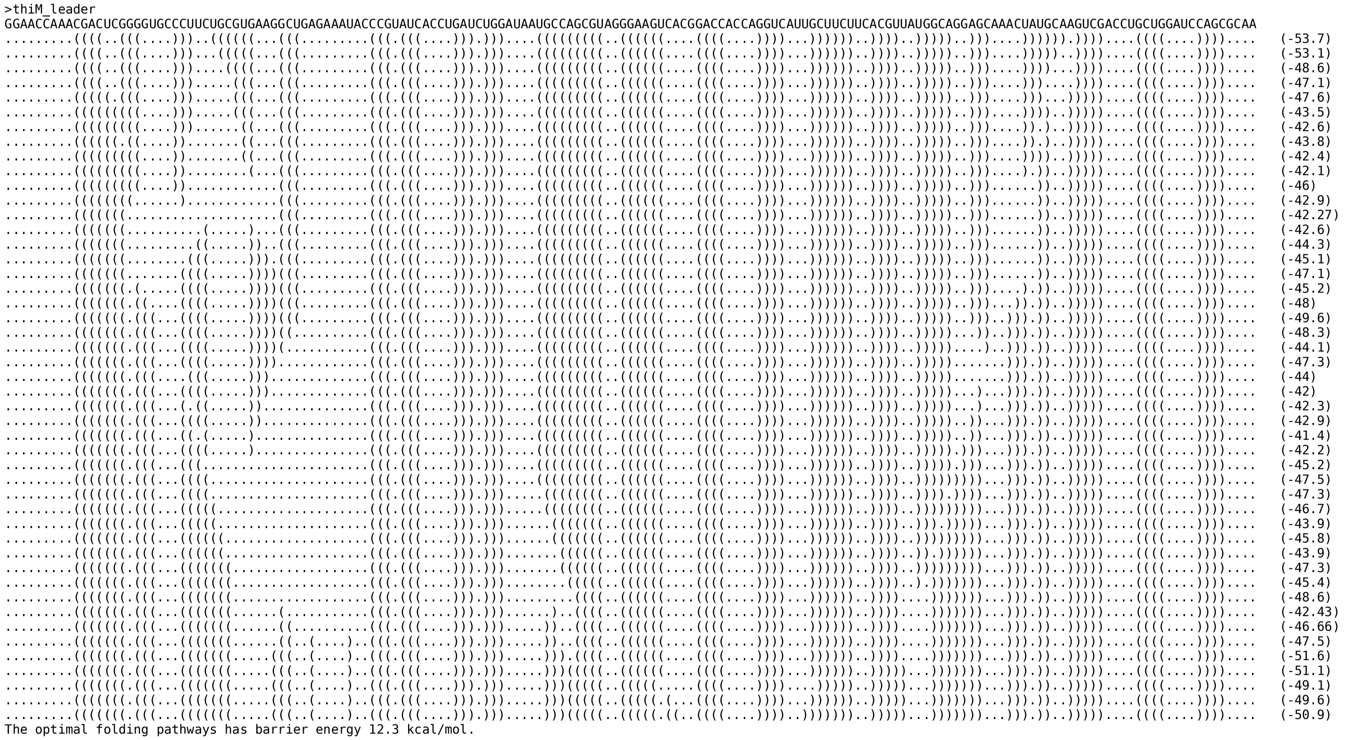

Figure 11: The best folding pathway between two conformational structures of thiM leader found by RNAEAPath over 5 runs using the default parameters.

```
>ms2
GGGUGGGACCCCUUUCGGGGUCCUGCUCAACUUCCUGUCGAGCUAAUGCCAUUUUUAAUGUCUUUAGCGAGAC
(((..(((((((....)))))))..)))........(((..(((((.(.(((.....))).).)))))..)))   (-32.7)
(((.((((((((....)))))))).)))........(((..(((((.(.(((.....))).).)))))..)))   (-29.3)
((((((((((((....))))))))))))........(((..(((((.(.(((.....))).).)))))..)))   (-32.6)
((((((((((((....))))))))))))........(((..(((((.(..((.....))..).)))))..)))   (-28.9)
((((((((((((....))))))))))))........(((..(((((.((.((.....))).).)))))..)))   (-28.8)
((((((((((((....))))))))))))........(((..(((((.((.(((...))))).).)))))..)))   (-28.8)
((((((((((((....))))))))))))........(((..(((((..(.(((...))))...)))))..)))   (-28)
((((((((((((....))))))))))))........(((..(((((.((.(((...)))))..)))))..)))   (-30)
((((((((((((....))))))))))))........(((..((((..((.(((...)))))...))))..)))   (-28.2)
((((((((((((....))))))))))))........(((..(((...((.(((...)))))....)))..)))   (-26.5)
((((((((((((....))))))))))))........(((..((((..((.(((...)))))..).)))..)))   (-26.1)
((((((((((((....))))))))))))........(((..(((((.((.(((...))))).)).)))..)))   (-27.3)
The optimal folding pathways has barrier energy 6.6 kcal/mol.
```

Figure 12: The best folding pathway between two conformational structures of ms2 found by RNAEAPath over 5 runs using the default parameters.

```
>HDV
GGAAUGGCGGGACCCCACUCUGCAGGGUCCGCGUUCCAUCCUUUCUUACCUGAUGGCCGGCAUGGUCCCAGCCUCCUCGCUGGCGCCGGCUGGGCAACAUUCCGAGGGGACCGUCCCCUCGGUAAUGGCGAAUGGGACCCACAAAUCUCUCUA
(((.(((.(....)))).)))...((((((.(((((.................((.(((((.((((.(((((......))))).))))))))).)).((((((((((((.....)))))))).))))..))))))))))).............   (-70.1)
.((.(((.(....)))).))....((((((.(((((.................((.(((((.((((.(((((......))))).))))))))).)).((((((((((((.....)))))))).))))..))))))))))).............   (-69)
..(.(((.(....)))).).....((((((.(((((.................((.(((((.((((.(((((......))))).))))))))).)).((((((((((((.....)))))))).))))..))))))))))).............   (-65.4)
....(((.(....)))).......((((((.(((((.................((.(((((.((((.(((((......))))).))))))))).)).((((((((((((.....)))))))).))))..))))))))))).............   (-67.4)
.....((.(....)))........((((((.(((((.................((.(((((.((((.(((((......))))).))))))))).)).((((((((((((.....)))))))).))))..))))))))))).............   (-66.7)
......(.(....)).........((((((.(((((.................((.(((((.((((.(((((......))))).))))))))).)).((((((((((((.....)))))))).))))..))))))))))).............   (-62.5)
........(....)..........((((((.(((((.................((.(((((.((((.(((((......))))).))))))))).)).((((((((((((.....)))))))).))))..))))))))))).............   (-63.3)
........................((((((.(((((.................((.(((((.((((.(((((......))))).))))))))).)).((((((((((((.....)))))))).))))..))))))))))).............   (-64.9)
.........................(((((.(((((.................((.(((((.((((.(((((......))))).))))))))).)).((((((((((((.....)))))))).))))..))))))))))..............   (-60.5)
..............(.........)(((((.(((((.................((.(((((.((((.(((((......))))).))))))))).)).((((((((((((.....)))))))).))))..))))))))))..............   (-55.4)
..............(.........).((((.(((((.................((.(((((.((((.(((((......))))).))))))))).)).((((((((((((.....)))))))).))))..)))))))))...............   (-53.4)
.............((.........))((((.(((((.................((.(((((.((((.(((((......))))).))))))))).)).((((((((((((.....)))))))).))))..)))))))))...............   (-55.4)
.............((.........)).(((.(((((.................((.(((((.((((.(((((......))))).))))))))).)).((((((((((((.....)))))))).))))..))))))))................   (-53.7)
............(((.........)))(((.(((((.................((.(((((.((((.(((((......))))).))))))))).)).((((((((((((.....)))))))).))))..))))))))................   (-55.9)
............(((.........))).((.(((((.................((.(((((.((((.(((((......))))).))))))))).)).((((((((((((.....)))))))).))))..))))))).................   (-55.2)
...........((((.........))))((.(((((.................((.(((((.((((.(((((......))))).))))))))).)).((((((((((((.....)))))))).))))..))))))).................   (-56.2)
...........((((.........)))).(.(((((.................((.(((((.((((.(((((......))))).))))))))).)).((((((((((((.....)))))))).))))..)))))).................. (-53.4)
...........((((.........))))...(((((.................((.(((((.((((.(((((......))))).))))))))).)).((((((((((((.....)))))))).))))..)))))...................   (-53.9)
.........(.((((.........)))))..(((((.................((.(((((.((((.(((((......))))).))))))))).)).((((((((((((.....)))))))).))))..)))))...................   (-53.3)
........((.((((.........)))))).(((((.................((.(((((.((((.(((((......))))).))))))))).)).((((((((((((.....)))))))).))))..)))))...................   (-57.6)
.......(((.((((.........)))))))(((((.................((.(((((.((((.(((((......))))).))))))))).)).((((((((((((.....)))))))).))))..)))))...................   (-58.2)
.......(((.((((.........))))))).((((.................((.(((((.((((.(((((......))))).))))))))).)).((((((((((((.....)))))))).))))..))))....................   (-56.1)
.....(.(((.((((.........))))))))((((.................((.(((((.((((.(((((......))))).))))))))).)).((((((((((((.....)))))))).))))..))))....................   (-55.1)
.....(.(((.((((.........)))))))).(((.................((.(((((.((((.(((((......))))).))))))))).)).((((((((((((.....)))))))).))))..)))).....................   (-55.3)
....((.(((.((((.........)))))))))(((.................((.(((((.((((.(((((......))))).))))))))).)).((((((((((((.....)))))))).))))..))).....................   (-54.8)
....((.(((.((((.........))))))))).((.................((.(((((.((((.(((((......))))).))))))))).)).((((((((((((.....)))))))).))))..))......................   (-54.7)
...(((.(((.((((.........))))))))))((.................((.(((((.((((.(((((......))))).))))))))).)).((((((((((((.....)))))))).))))..))......................   (-55.5)
...(((.(((.((((.........)))))))))).(.................((.(((((.((((.(((((......))))).))))))))).)).((((((((((((.....)))))))).))))..).......................   (-54)
..((((.(((.((((.........)))))))))))(.................((.(((((.((((.(((((......))))).))))))))).)).((((((((((((.....)))))))).))))..).......................   (-54.9)
..((((.(((.((((.........)))))))))))..................((.(((((.((((.(((((......))))).))))))))).)).((((((((((((.....)))))))).))))..........................   (-57.4)
.(((((.(((.((((.........))))))))))))).................((.(((((.((((.(((((......))))).))))))))).)).((((((((((((.....)))))))).))))..........................   (-60.6)
((((((.(((.((((.........)))))))))))))................((.(((((.((((.(((((......))))).))))))))).)).((((((((((((.....)))))))).))))..........................   (-64.8)
((((((.(((.((((.........))))))))))))).......(........((.(((((.((((.(((((......))))).))))))))).)).((((((((((((.....)))))))).))))..).......................   (-62.9)
((((((.(((.((((.........)))))))))))))......((........((.(((((.((((.(((((......))))).))))))))).)).((((((((((((.....)))))))).))))..))......................   (-64.6)
((((((.(((.((((.........))))))))))))).....(((........((.(((((.((((.(((((......))))).))))))))).)).((((((((((((.....)))))))).))))..))).....................   (-65.3)
((((((.(((.((((.........)))))))))))))....((((........((.(((((.((((.(((((......))))).))))))))).)).((((((((((((.....)))))))).))))..))).)...................   (-62.2)
((((((.(((.((((.........)))))))))))))...(((((........((.(((((.((((.(((((......))))).))))))))).)).((((((((((((.....)))))))).))))..))).))..................   (-65.5)
((((((.(((.((((.........)))))))))))))..((((((........((.(((((.((((.(((((......))))).))))))))).)).((((((((((((.....)))))))).))))..))).))).................   (-68.4)
((((((.(((.((((.........))))))))))))).(((((((........((.(((((.((((.(((((......))))).))))))))).)).((((((((((((.....)))))))).))))..))).))))................   (-69.6)
((((((.(((.((((.........))))))))))))).(((((((........((.(((((.((((.(((((......))))).))))))))).)).(.((((((((((.....)))))))).)).)..))).))))................   (-65.3)
((((((.(((.((((.........))))))))))))).(((((((........((.(((((.((((.(((((......))))).))))))))).)).((((((((((((.....)))))))).))))..))).))))................   (-69.6)
The optimal folding pathways has barrier energy 16.8 kmol/cal.
```

Figure 13: The best folding pathway between two conformational structures of HDV found by RNAEAPath over 5 runs using the default parameters.

```
>dsrA
ACACAUCAGAUUUCCUGGUGUAACGAAUUUUUUAAGUGCUUCUUGCUUAAGCAAGUUUCAUCCCGACCCCCUCAGGGUCGGGAUU
..(((((((.....)))))))...(((((((((((((.......))))))).)))))).((((((((((.....))))))))))).   (-32.3)
..(((((((.....)))))))...((((((((((((.........)))))).)))))).((((((((((.....))))))))))).   (-30.8)
..(((((((.....)))))))...((((((((((((..(.....))))))).)))))).((((((((((.....))))))))))).   (-28.6)
..(((((((.....)))))))...(((((((((((...(.....).))))).)))))).((((((((((.....))))))))))).   (-25.9)
..(((((((.....)))))))...(((((((((((..((.....))))))).)))))).((((((((((.....))))))))))).   (-29.4)
..(((((((.....)))))))....((((((((((..((.....))))))).)))))..((((((((((.....))))))))))).   (-28.3)
..(((((((.....))))))).....(((((((((..((.....))))))).))))...((((((((((.....))))))))))).   (-27.3)
..(((((((.....)))))))......((((((((..((.....))))))).)))....((((((((((.....))))))))))).   (-26.4)
..(((((((.....))))))).......(((((((..((.....))))))).)).....((((((((((.....))))))))))).   (-25.9)
..(((((((.....)))))))........((((((..((.....))))))).)......((((((((((.....))))))))))).   (-25)
..(((((((.....))))))).........(((((..((.....))))))).......((((((((((.....))))))))))).   (-27.2)
..(((((((.....))))))).........((((...((.....)).)))).......((((((((((.....))))))))))).   (-26.9)
..(((((((.....)))))))..........(((...((.....)).))).........((((((((((.....))))))))))).   (-26.6)
..(((((((.....)))))))...........((...((.....)).))..........((((((((((.....))))))))))).   (-25.7)
..(((((((.....)))))))............(...((.....)).)...........((((((((((.....))))))))))).   (-24.3)
..(((((((.....)))))))................((.....))..............((((((((((.....))))))))))).   (-27.8)
..(((((((.....)))))))................(.......)..............((((((((((.....))))))))))).   (-24.5)
..(((((((.....))))))).......................................((((((((((.....))))))))))).   (-26.8)
..(((((((.....)))))))......................(......).......((((((((((.....))))))))))).   (-25.5)
..(((((((.....))))))).....................((......)).......((((((((((.....))))))))))).   (-26.4)
..(((((((.....)))))))....................(.((......)).)....((((((((((.....))))))))))).   (-25.5)
..(((((((.....)))))))....................(.(((....))).)....((((((((((.....))))))))))).   (-28)
..(((((((.....)))))))....................(((((....)))))....((((((((((.....))))))))))).   (-32.1)
..(((((((.....)))))))................(...(((((....)))))...)((((((((((.....))))))))))).   (-29.8)
The optimal folding pathways has barrier energy 8 kcal/mol.
```

Figure 14: The best folding pathway between two conformational structures of dsrA found by RNAEAPath over 5 runs using the default parameters.

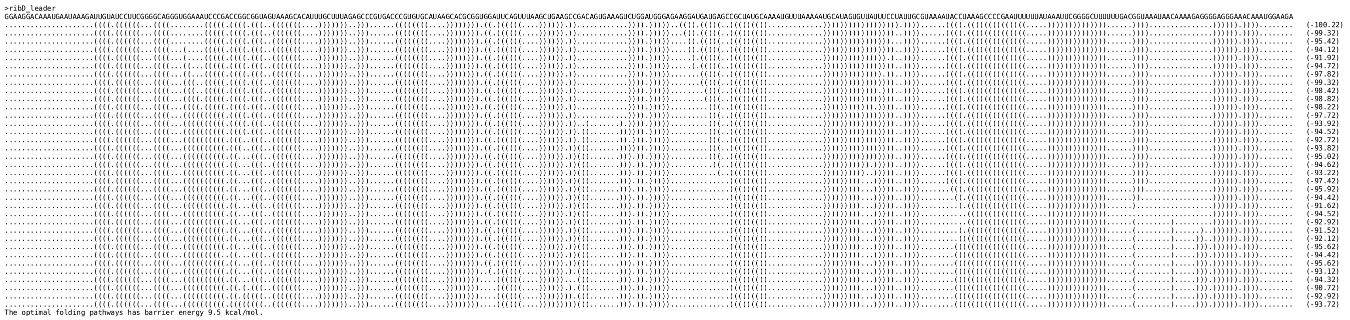

Figure 15: The best folding pathway between two conformational structures of ribD leader found by RNAEAPath over 5 runs using the default parameters.

```
>amv
AAUGCUGACCUCCACUGGGUGGAUUAAGGUCAAGGUAUGAAGUCCUAUUCGCUCCUGAUAGGAUCGACUUCAUAUUGCUUAUAUAUGUGCUAACGCACAUAUAUAAAUGCUCAUGCAAAACUGCAUGAAUGCCCCUAAGGGAUGC
.....((((((((((...)))))....))))).((((((((((((((((.......)))))))....)))))))))..((((((((((((....))))))))))))....(((((((....)))))))...(((....)))....   (-52)
.....((((((((((...)))))....))))).((((((((((.(((((.......)))))).....)))))))))..((((((((((((....))))))))))))....(((((((....)))))))...(((....)))....   (-49.7)
.....((((((((((...)))))....))))).((((((((((((((((.......))))))....))))))))))..((((((((((((....))))))))))))....(((((((....)))))))...(((....)))....   (-51.8)
.....((((((((((...)))))....))))).((((((((((.(((((.......))))).....))))))))))..((((((((((((....))))))))))))....(((((((....)))))))...(((....)))....   (-47.9)
.....((((((((((...)))))....))))).((((((((((((((((.......)))))....)))))))))))..((((((((((((....))))))))))))....(((((((....)))))))...(((....)))....   (-51.4)
.....((((((((((...)))))....))))).(((((((((((((((.........))))....)))))))))))..((((((((((((....))))))))))))....(((((((....)))))))...(((....)))....   (-50.1)
.....((((((((((...)))))....))))).((((((((((((((...........)))....)))))))))))..((((((((((((....))))))))))))....(((((((....)))))))...(((....)))....   (-48.6)
.....((((((((((...)))))....))))).(((((((((((((.............))....)))))))))))..((((((((((((....))))))))))))....(((((((....)))))))...(((....)))....   (-47.32)
.....((((((((((...)))))....))))).((((((((((((...............)....)))))))))))..((((((((((((....))))))))))))....(((((((....)))))))...(((....)))....   (-46.26)
.....((((((((((...)))))....))))).(((((((((((.....................)))))))))))..((((((((((((....))))))))))))....(((((((....)))))))...(((....)))....   (-48.7)
.....((((((((((...)))))....))))).(((((((((((.....(..........)....)))))))))))..((((((((((((....))))))))))))....(((((((....)))))))...(((....)))....   (-47.9)
.....((((((((((...)))))....))))).(((((((((((....((..........))...)))))))))))..((((((((((((....))))))))))))....(((((((....)))))))...(((....)))....   (-48.9)
.....((((((((((...)))))....))))).(((((((((((...(((..........)))..)))))))))))..((((((((((((....))))))))))))....(((((((....)))))))...(((....)))....   (-50.6)
.....((((((((((...)))))....))))).(((((((((((...(((.(....)...)))..)))))))))))..((((((((((((....))))))))))))....(((((((....)))))))...(((....)))....   (-50.8)
.....((((((((((...)))))....))))).(((((((((((...(((.(....)...)))..)))))))))))..((((((((((((....))))))))))))..(.(((((((....)))))))...(((....)))...)   (-46.7)
.....((((((((((...)))))....))))).(((((((((((...(((.(....)...)))..)))))))))))..((((((((((((....))))))))))))..(((((((((....)))))))...(((....)))..))   (-49.5)
.....((((((((((...)))))....))))).(((((((((((..((((.(....)...)))).)))))))))))..((((((((((((....))))))))))))..(((((((((....)))))))...(((....)))..))   (-50.6)
......(((((((((...)))))....))))..(((((((((((..((((.(....)...)))).)))))))))))..((((((((((((....))))))))))))..(((((((((....)))))))...(((....)))..))   (-50.1)
....(.(((((((((...)))))....))))..(((((((((((..((((.(....)...)))).))))))))))).((((((((((((....))))))))))))..(((((((((....)))))))...(((....)))..))   (-46.5)
...((.(((((((((...)))))....))))..(((((((((((..((((.(....)...)))).)))))))))))))((((((((((((....))))))))))))..(((((((((....)))))))...(((....)))..))   (-49.3)
...((((((((((((...)))))....))))).(((((((((((..((((.(....)...)))).)))))))))))))((((((((((((....))))))))))))..(((((((((....)))))))...(((....)))..))   (-48.5)
The optimal folding pathways has barrier energy 5.74 kcal/mol.
```

Figure 16: The best folding pathway between two conformational structures of amv found by RNAEAPath over 5 runs using the default parameters.

```
>alpha_operon
GUCUGUGCGUUUCCAUUUGAGUAUCCUGAAAACGGGCUUUUCAGCAUGGAACGUACAUAUUAAAUAGUAGGAGUGCAUAGUGGCCCGUAUAGCAGGCAUUAACAUUCCUGAUCAUAAGCAUGCCGUAAUC
...((((((((.(((((((((...((((....))))...))))).))))))))))))..........((((((((..(((((.((.(.....).))))))).))))))))....................   (-37.1)
...((((((((.(((((((((...((((....))))...))))).))))))))))))..........((((((((..(((.(.((.(.....).))).))).))))))))....................   (-32.5)
...((((((((.(((((((((...((((....))))...))))).))))))))))))..........((((((((..(((...((.(.....).))..))).))))))))....................   (-31)
...((((((((.(((((((((...((((....))))...))))).))))))))))))..........((((((((..(((..(((.(.....).))).))).))))))))....................   (-32.6)
...((((((((.(((((((((...((((....))))...))))).))))))))))))..........((((((((..((((.(((.(.....).))))))).))))))))....................   (-37.1)
...(((((((..(((((((((...((((....))))...))))).)))).)))))))..........((((((((..((((.(((.(.....).))))))).))))))))....................   (-32.8)
...(((((((.((((((((((...((((....))))...))))).))))))))))))..........((((((((..((((.(((.(.....).))))))).))))))))....................   (-37.1)
...(((((((.((((((((((...((((....))))...))))).))))))))))))..........((((((((..((((.(((.((...)).))))))).))))))))....................   (-36.8)
...((((((..((((((((((...((((....))))...))))).))))).))))))..........((((((((..((((.(((.((...)).))))))).))))))))....................   (-32.7)
...((((((.(((((((((((...((((....))))...))))).))))))))))))..........((((((((..((((.(((.((...)).))))))).))))))))....................   (-36.8)
...((((((.(((((((((((...((((....))))...))))).))))))))))))...........(((((((..((((.(((.((...)).))))))).))))))).....................   (-35.7)
..(((((((.(((((((((((...((((....))))...))))).))))))))))))...........(((((((..((((.(((.((...)).))))))).))))))))....................   (-31.5)
.((((((((.(((((((((((...((((....))))...))))).))))))))))))...........(((((((..((((.(((.((...)).))))))).)))))))))...................   (-33)
(((((((((.(((((((((((...((((....))))...))))).))))))))))))...........(((((((..((((.(((.((...)).))))))).))))))))))..................   (-33.7)
The optimal folding pathways has barrier energy 6.1 kcal/mol.
```

Figure 17: The best folding pathway between two conformational structures of alpha operon found by RNAEAPath over 5 runs using the default parameters.

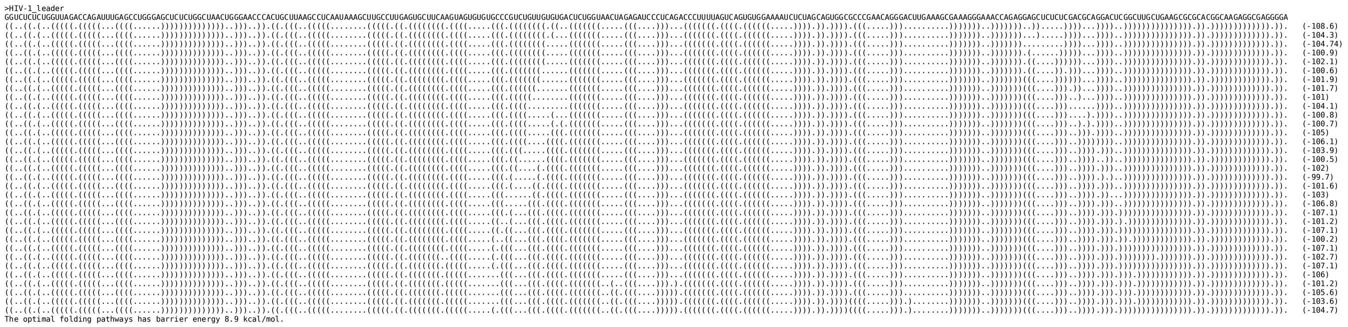

Figure 18: The best folding pathway between two conformational structures of HIV-1 leader found by RNAEAPath over 5 runs using the default parameters.
